# My Life, My Story: Integrating a Life Story Narrative Component Into Medical Student Curricula

**DOI:** 10.15766/mep_2374-8265.11211

**Published:** 2022-01-26

**Authors:** Jeffrey A. Lam, Mara Feingold-Link, Julia Noguchi, Anne Quinn, Dana Chofay, Kate Cahill, Steven Rougas

**Affiliations:** 1 Bray Humanities Fellow, Warren Alpert Medical School of Brown University; 2 Fellow, Department of Palliative Care, Warren Alpert Medical School of Brown University; 3 Director of Community Engagement and Scholarship, Doctoring Program, Warren Alpert Medical School of Brown University; Doctor of Public Health Candidate, Department of Community Health Sciences, Boston University School of Public Health; 4 My Life, My Story Volunteer Coordinator, Warren Alpert Medical School of Brown University; 5 Doctoring Program Course Leader and Clinical Assistant Professor of Medicine and Medical Sciences, Warren Alpert Medical School of Brown University; 6 Internal Medicine Clerkship Director, Associate Professor of Medicine and Medical Sciences, and Clinician Educator, Warren Alpert Medical School of Brown University; 7 Doctoring Program Director and Associate Professor of Emergency Medicine and Medical Sciences, Warren Alpert Medical School of Brown University

**Keywords:** Patient-Centered Care, Well-Being/Mental Health, Medical Humanities, Reflection/Narrative Medicine

## Abstract

**Introduction:**

Medical students experience burnout, depersonalization, and decreases in empathy throughout medical training. My Life, My Story (MLMS) is a narrative medicine project that aims to combat these adverse outcomes by teaching students to interview patients about their life story, with the goal of improving patient-centered care competencies, such as empathy.

**Methods:**

The MLMS project was started in the Veterans Affairs (VA) system and has since spread to dozens of VA sites. We adapted and integrated this project into the Warren Alpert Medical School of Brown University curriculum. As part of the required curriculum, first- and third-year medical students participated in a life story interview with a community-based volunteer or a patient in the inpatient hospital setting, transcribed the story, and reviewed the written story with the patient. We assessed student perceptions of the project, changes in empathy, and changes in burnout symptoms.

**Results:**

A total of 240 students participated in this project. Students spent an average of 70.7 minutes interviewing patients. A majority of the students believed MLMS was a good use of time (77%), fostered connection with patients (79%), and was effective in recognizing patients’ thoughts and feelings (69%).

**Discussion:**

To our knowledge, this is one of the first life story interview interventions to be implemented into a required medical school curriculum and outside the VA setting. MLMS may assist students in improving clinical empathy skills and create a structure for medical trainees to better understand their patients.

## Educational Objectives

By the end of this activity, learners will be able to:

1. Apply patient-centered care competencies when obtaining a patient's life story.
2. List the patient's strengths and values by writing their life story in their own words.
3. Identify the complex physical, mental, social, and environmental factors that contribute to well-being over a patient's life span.
4. Describe how longitudinal relationships can contribute to patient care.
5. Practice empathetic, nonjudgmental listening skills.

## Introduction

The term *compassion crisis* has been used to describe the lack of patient-centered skills in our health care system.^1^ A patient survey conducted in 2011 found nearly half the patients believed the American health care system is not compassionate.^2^ There are numerous reports of empathy decreasing over the course of medical training,^3^ and a majority of health care providers do not believe they have sufficient time to demonstrate empathy.^4^ Furthermore, physicians listen to their patients for an average of 11 seconds before interrupting,^5^ and audiotaped routine office visits indicate that less than 1% of physicians use expressions of empathy or compassion.^6^

Patient-centered care (PCC), defined as care that is respectful and responsive to patient preferences and values, may combat these worrying trends. PCC improves outcomes for both patients and providers. Multiple meta-analyses demonstrate that PCC is associated with improved patient outcomes, anxiety, and distress.^7^ From a provider perspective, building compassion may improve provider depression, anxiety, and overall well-being.^8^ PCC competencies, such as empathy, are inversely associated with burnout,^9^ a syndrome of emotional exhaustion, depersonalization, and lack of personal accomplishment.

PCC improves with practice and training.^10^ Specifically, narrative medicine interventions have been associated with enhanced PCC competencies, increased empathy, and reduced emotional exhaustion for health care workers.^11,12^ My Life, My Story (MLMS) is a narrative medicine educational initiative that invites patients to share their life experience with their health care team through a structured life history interview^13^ and incorporation of a written life narrative into the medical chart. The Veterans Affairs (VA) system started the MLMS project in 2013, and the project has since spread to dozens of VA sites across the country. MLMS has been demonstrated to help improve trainees’ PCC competencies, including empathy,^14^ and has strong long-term acceptability in VA settings.^15^ From a patient perspective, reminiscence interventions have demonstrated meaningful increases in mental health^16^ and overall well-being outcomes^17^ in numerous populations, including patients with dementia,^18^ patients with cancer,^19^ and geriatric patients.^20^ From a provider perspective, including patient narratives in the electronic medical record has fostered improved communication and a feeling of increased connection,^21^ and the majority of providers agree that reading these narratives allows them to take better care of their patients.^13^

While other sites have built MLMS into the expectations of rotating trainees or developed volunteer programs,^14,22,23^ there are no published reports of life review interventions implemented (1) in a non-VA setting or (2) into required undergraduate medical education (UME) curricula at multiple training levels. The goals of this resource are to (1) disseminate curricular materials for MLMS as designed for UME and (2) demonstrate the acceptability and effect of the MLMS project in required curricula on PCC competencies and empathy at the Warren Alpert Medical School (AMS) of Brown University.

## Methods

### Overview

In consultation with AMS curriculum leaders, MLMS was integrated into two parts of the mandatory curriculum for the 2020–2021 academic year: (1) First-year medical students completed an MLMS interview via phone or online videoconference with an older adult volunteer recruited from a community-based organization as part of their required clinical skills doctoring course, and (2) third-year medical students completed an MLMS interview with an inpatient during their internal medicine core clerkship rotation. The development of our curriculum was modeled on previous MLMS projects implemented at other institutions, and we adapted materials from the national VA MLMS organization.^24^ Our materials differed from the VA MLMS project in two important ways: (1) The materials included an interview question guide tailored to a general patient population (as opposed to a veteran population), and (2) our facilitation guides focused on UME learning and included educational objectives, suggested readings, references, and a structure for debriefing the experience. The primary steps of the project remained largely the same as the VA MLMS project. Working with our technological officers within our affiliated training institutions, we integrated MLMS narratives into Epic, the electronic health record (EHR) at the two largest training sites. One of our project team members had previously assisted with the implementation of an MLMS project at another academic medical institution, which provided our team essential information on navigating the implementation process.

### Implementation

All students took part in a virtual 45-minute training session facilitated by our team before conducting MLMS interviews (Appendix A). This training session presented students with a theoretical framework for the intervention and the significance of patient narratives, as well as procedural information on best practices for asking a patient questions about their life story, writing a 1,000-word narrative, reviewing the story with the patient, and (for third-year students only) integrating the story into the EHR. Students received a facilitation guide containing detailed information on the expectations and logistics to complete the MLMS interview as well as suggested interview questions, adopted from the national VA MLMS resources. First-year students completed the project over a 4-week period over the phone or videoconferencing technology with an older adult volunteer in the community. Our MLMS volunteer coordinator recruited volunteers from 16 local community organizations. Each community organization had an identified point of contact, and all of the volunteers were provided with a program description. We randomly assigned each first-year medical student to a community volunteer, except when there was a specific language need. We provided the first-year students with the volunteer's name and contact information. This process included an introductory phone call, a life story interview, review of the story, and a written reflection on the experience (Appendix B). The written reflections were not included as part of our research evaluation. At the end of the project, first-year students debriefed their experiences in an in-person small-group session (eight to 10 students) facilitated by the doctoring course faculty members. In conjunction with a debrief guide (Appendix B), faculty used students’ written reflections as a starting point for the conversation.

Third-year students had 3 weeks to complete the MLMS process during their internal medicine core clerkship rotation. This process included identifying an interested patient, conducting a life story interview in person at the bedside, reviewing the story with the patient, and submitting the story for inclusion in the electronic medical record (Appendix C). After completing the MLMS project, these third-year students participated in a virtual, small-group (about 25 students) debrief session led by two facilitators. The first facilitator had led and participated in the MLMS program at Brigham and Women's Hospital as a resident physician. While she was a hospice and palliative care fellow at Brown, she trained the second facilitator, a medical student participating in a humanities fellowship between his third and fourth years of medical school.

### Evaluation

During both the training and debrief sessions, we emailed all first- and third-year students an invitation to complete an optional baseline and then postintervention survey (Appendix D). While participation in this project was required as part of the curriculum, the pre- and postsurvey evaluations were optional. Students who participated in our research surveys completed an online informed consent form regarding their participation in our survey research, which was approved by the Brown University Institutional Review Board.

Consistent with previous interventions,^14^ we adapted the consultation and relationship empathy (CARE) measure^25^ to measure PCC competencies believed to be imparted by the MLMS program and assessed changes in empathy from baseline to postintervention. We also assessed the direct impact of the MLMS program for these same PCC competencies. We used five items from the CARE measure that evaluated self-reports of clinical empathy skills such as making the patient feel at ease, showing care and compassion, and being interested in the patient as a whole person. Each item was scored on a 5-point Likert scale (1 = *poor,* 5 = *excellent*). To measure the perceived effect of this project, we asked patients similar questions about whether the MLMS project was effective in changing these skills (1 = *not effective at all,* 5 = *extremely effective*). For this analysis, the baseline CARE measure had an alpha of .89.

We also assessed changes in burnout symptoms from baseline to postintervention among the third-year clerkship students using an adapted and abbreviated Maslach Burnout Inventory (aMBI).^26,27^ We omitted giving the burnout questions to the first-year students because (1) we did not hypothesize that the intervention would lead to meaningful decreases in burnout symptoms this early in UME and (2) we wanted to limit the size of our survey to reduce survey fatigue. The aMBI had three domains: (a) emotional exhaustion, (b) depersonalization, and (c) personal accomplishment. Our aMBI was scored on a 7‐point Likert scale (0 = *never,* 6 = *every day*), and three items were reverse-scored in order to create an average burnout item score for baseline and postintervention. For this analysis, the baseline aMBI measure had an alpha of .66.

Lastly, we assessed students’ overall perception of the MLMS program through qualitative and quantitative survey responses. Participants were asked to give open responses to the following questions:

• How was your experience?
• What, if any, is the value of this project?
• Do you have any suggestions for how we could improve this project in the future?

Participants were also asked to rate their agreement with statements related to this project such as “This project was a good use of time” and “This experience fostered more connection with patients” on a 5-point Likert scale (1 = *strongly disagree,* 5 = *strongly agree*).

### Data Analysis

We qualitatively analyzed open-ended responses using a constructivist grounded-theory approach to code each response. The research team explored connections between codes and refined categories to aggregate data into themes.

Descriptive analyses (means, standard deviations, and percentages) were used to describe implementation characteristics, trainee ratings of PCC competencies, perceived improvements in PCC competencies, aMBI scores, and student perceptions of the project. For the main analysis, we measured the difference in scores within participants between baseline and postintervention using paired *t* tests for the dependent variables of interest: PCC competencies and aMBI. Exploratory analyses were conducted to examine the relationships between average aMBI item score and time spent interviewing the patient for the third-year students. Analyses were performed in SPSS Version 26.

## Results

A total of 240 students took part in the initial implementation of the project: 146 (out of 146 students enrolled) from the first-year class, and 94 (out of 140 students enrolled) from the third-year class. We analyzed the data in February 2021, and thus, some of the third-year students had yet to complete the project. Out of the 146 first-year students, 66 (45%) completed a baseline survey, 62 (42%) completed a postintervention survey, and 29 (20%) completed both surveys. Out of the 94 third-year students, 75 (80%) completed a baseline survey, 52 (55%) completed a postintervention survey, and 30 (32%) completed both surveys. Students spent an average of 70.7 minutes interviewing the patient, with first-year students spending a significantly greater amount of time (*M* = 81.6, *SD* = 39.3) compared to third-year students (*M* = 55.4, *SD* = 32.6), *t*(123) = 4.0, *p* ≤ .001.

Out of the 114 students who completed a postsurvey, 83 commented on the overall experience, 76 commented on the value of the experience, and 48 commented with suggestions. Narrative comments from these three questions were organized into three themes: (1) perceived benefits for students, (2) perceived benefits for patients, and (3) factors influencing the experience. In the factors that influenced this intervention, first-year students commented on the patient they were assigned to interview, while third-year students commented on perceived time available. Table 1 presents qualitative results from the open-response questions.

**Table 1.**
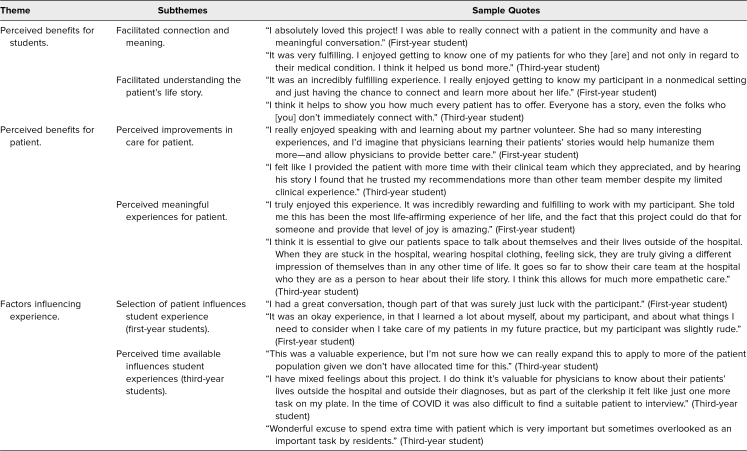
Summary of Qualitative Responses

Table 2 presents the students’ perceptions of the MLMS program: Eighty-nine out of 116 (77%) stated that they somewhat or strongly agreed that the project was a good use of time, 93 out of 116 (80%) agreed it was a valuable experience, 91 out of 115 (79%) agreed it fostered connection with patients, 94 out of 115 (82%) agreed having life story interviews with patients might improve patient care, and 91 out of 115 (79%) agreed it was an experience not provided in other aspects of their medical education. Two-sample *t* tests indicated no differences in these ratings between first- and third-year students, with the exception that first-year students (*M* = 4.4, *SD* = 0.8) indicated more than third-year students (*M* = 3.9, *SD* = 1.3) that this experience was not offered anywhere else in their medical education curriculum, *t*(112) = 2.5, *p* = .02.

**Table 2.**
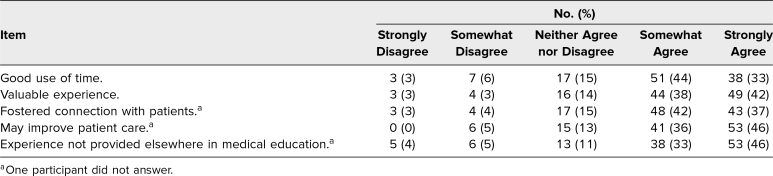
Student Perception of My Life, My Story Project (*N* = 116)

Tables 3 and 4 present paired *t* tests comparing the baseline and postintervention surveys and self-rated improvements in PCC competencies. Paired *t* tests found higher ratings on the postsurvey in all five PCC competencies compared to the baseline surveys. Similarly, students indicated that they believed the intervention improved their PCC competencies, with a majority of students indicating it was moderately to extremely effective in recognizing the patient's thoughts and feelings (81 out of 117, 69%), being attentive and responsive (82 out of 115, 71%), treating the patient in a caring manner (84 out of 116, 72%), displaying empathy (86 out of 116, 74%), and showing genuine interest (93 out of 116, 80%). There were no significant differences between perceived improvements in PCC competencies between first- and third-year students.

**Table 3.**
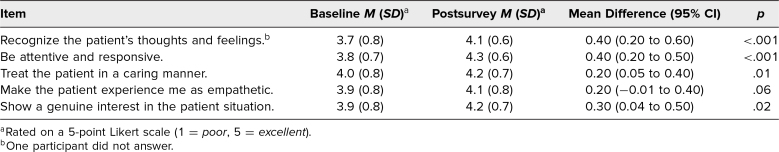
Changes in Self-Rated Clinical Empathy Skills (*N* = 60)

**Table 4.**
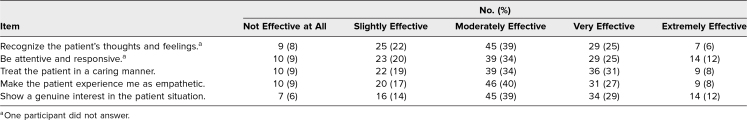
Perceived Effect of Intervention on Self-Rated Clinical Empathy Skills (*N* = 116)

Overall, there was no significant difference in scores in the average aMBI burnout from baseline (*M* = 3.3, *SD* = 0.9) to postintervention (*M* = 3.4, *SD* = 1.0) for the third-year students, *t*(30) = 0.2, *p* = .84. Results of the Spearman correlation indicated that there was a significant negative association between average aMBI item score and minutes spent interviewing the patient, *r*s(28) = −.42, *p* = .02.

## Discussion

We modified and implemented a life story review intervention across the AMS curriculum, with a goal of teaching students PCC competencies, such as empathy. To our knowledge, this is one of the first life review interventions implemented into required curricula across multiple training sites for learners at various levels, as well as the first time that results from the MLMS project have been replicated and published in a non-VA setting. These are the first peer-reviewed MLMS materials to be tailored to the UME level. Students had positive perceptions of the MLMS program, and the intervention improved self-perceived clinical empathy skills.

### Strengths

Our results suggest that a single MLMS interview improves self-perceived empathy levels for medical students, adding to the growing body of evidence suggesting empathy is modifiable.^8^ Given recent reports of empathy decreasing throughout UME^3^ and concerns about a compassion crisis in our health care system,^1^ MLMS may be one tangible approach to addressing these concerns.

Student perceptions of how MLMS affected clinical empathy skills did not significantly differ between the first- and third-year student programs, despite differences in student orientation training modality (in person vs. videoconference), patient recruitment methods (recruited from community vs. inpatient setting), and patient interview method (phone or online video conference technology vs. in person). The total implementation process took less than a year, including securing support of curricular leaders, integrating the stories into the largest training sites’ EHRs, and evaluating the project. AMS funded one clinical year medical student to coordinate and initiate the program but had few other resource requirements, especially once the program was initiated. MLMS is a practical and adaptable medical education module that can be implemented within preclinical coursework, core clerkships, and elective courses.

### Barriers

This project was not without barriers to implementation. While we were able to integrate the MLMS project into our primary training site's EHR within weeks, it took months to integrate the MLMS into other affiliate institution's EHRs. Another impediment to implementation is buy-in from educators, hospital leaders, and students. Addressing this barrier requires research that demonstrates MLMS has an impact on patient care and learner wellness.

### Lessons Learned

In the cohort of third-year students, burnout rates remained approximately the same from the presurvey to the postsurvey. Given the lack of a control group in our experimental design and the fact that the MLMS project was a proportionally small curricular component of the overall internal medicine rotation, we could not determine the direct effects of the MLMS project on burnout rates. Our exploratory analyses showed spending time interviewing patients about their life story was associated with a decreased risk of becoming more burned out during this clinical rotation. These results may be consistent with other reports finding that narrative medicine interventions may reduce emotional exhaustion amongst trainees.^11^ However, we caution against overinterpretation of this result, as this correlation could have been spurious given that we identified this finding post hoc. Future studies should further examine the effect of humanistic interventions on burnout given a lack of evidence-based, patient-focused curricular interventions intended to combat burnout.^28^

Students’ perceived time and the availability of interested patients influenced the students' perception of this project. Qualitative comments and anecdotal feedback indicated that third-year students identified time available as a significant factor influencing their experience with the MLMS project, as they were expected to find time inside of their internal medicine clerkship rotation to interview the patient and write up the story. While the third-year students spent less time interviewing the patients than the first-year students, the first-year students had more flexibility in their schedules as they were asked to call their patient on their own time. Given that first-year students were assigned to their patient, qualitative comments indicated students thought patients that they were matched with likely influenced their overall perception of the program, giving evidence that selection and engagement of patients were important factors in the program.

### Limitations

One limitation was that our intervention did not contain a control group for comparison. Our data were also limited by response bias, as only 141 out of 240 students (59%) responded to the baseline survey, 114 out of 240 (48%) to the postintervention survey, and 59 out of 240 (25%) to both surveys. The response rates may have differed because of the format in which the introductory and closing sessions were delivered; the third-year students were divided into five cohorts throughout the year, while the first-year students received the introductory information as a large group. These low response rates greatly limited the ability to quantitatively evaluate this intervention. While the surveys were based on participant self-report, the results were also longitudinal and asked participants how this particular curricular innovation changed the outcomes of interest. Lastly, given that our survey was sent out immediately postintervention, there were no data on how a single life story interview could improve any of the outcomes of interest longitudinally.

### Conclusion

In conclusion, MLMS was successfully implemented for two cohorts of medical students at different curricular levels, demonstrating the feasibility of integrating a life story interview into medical curricula. MLMS may allow students to improve clinical empathy skills, mitigate burnout symptoms, and understand patients as people.

## Appendices


PowerPoint Presentation.pptxPreclinical Facilitation Guide.docxClinical Facilitation Guide.docxSurvey Instruments.docx
*All appendices are peer reviewed as integral parts of the Original Publication.*
