# Supplementary material for: My Life, My Story: Integrating a Life Story Narrative Component Into Medical Student Curricula
Source: MedEdPORTAL. 2022 Jan 26;18:11211. doi: 10.15766/mep_2374-8265.11211 (PMC8789965; doi:10.15766/mep_2374-8265.11211)
Supplement: Supplementary file 1 — PowerPoint Presentation.pptxPreclinical Facilitation Guide.docxClinical Facilitation Guide.docxSurvey Instruments.docx [file mep_2374-8265.11211-s001.zip › A. Powerpoint Presentation.pptx]

## Slide 1
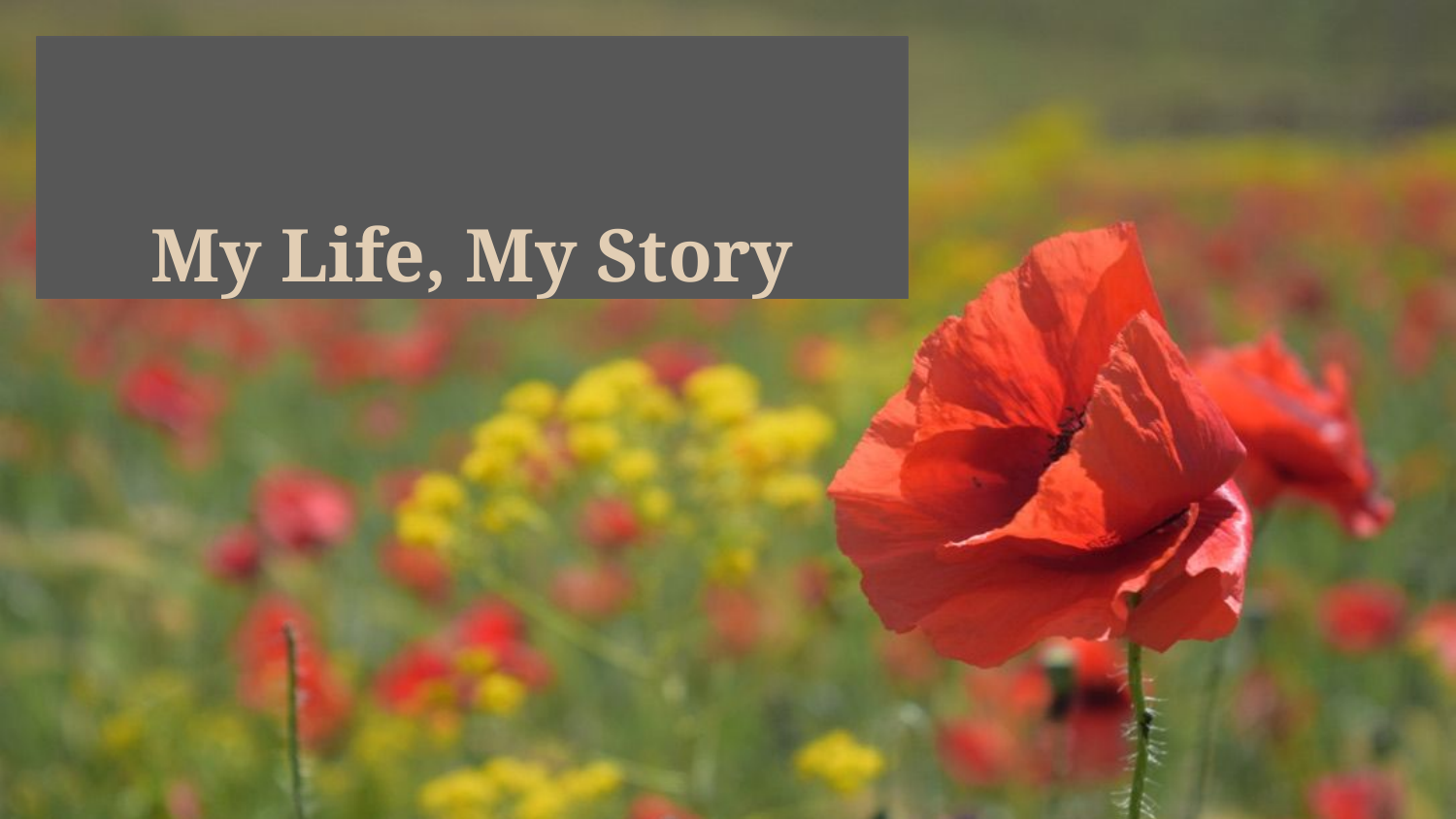

My Life, My Story

## Slide 2
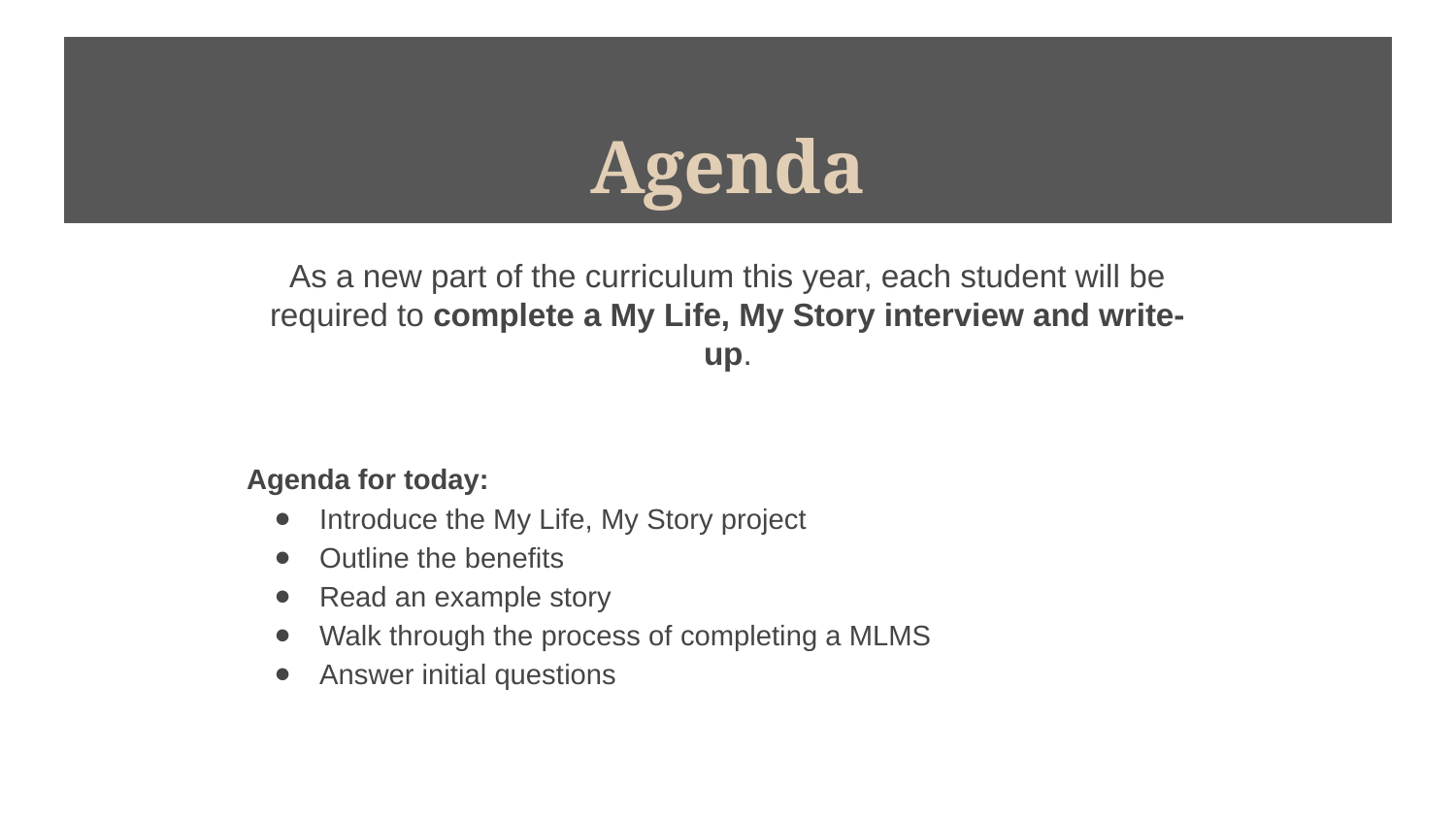

Agenda
As a new part of the curriculum this year, each student will be required to complete a My Life, My Story interview and write-up.
Agenda for today:
Introduce the My Life, My Story project
Outline the benefits
Read an example story
Walk through the process of completing a MLMS
Answer initial questions

## Slide 3
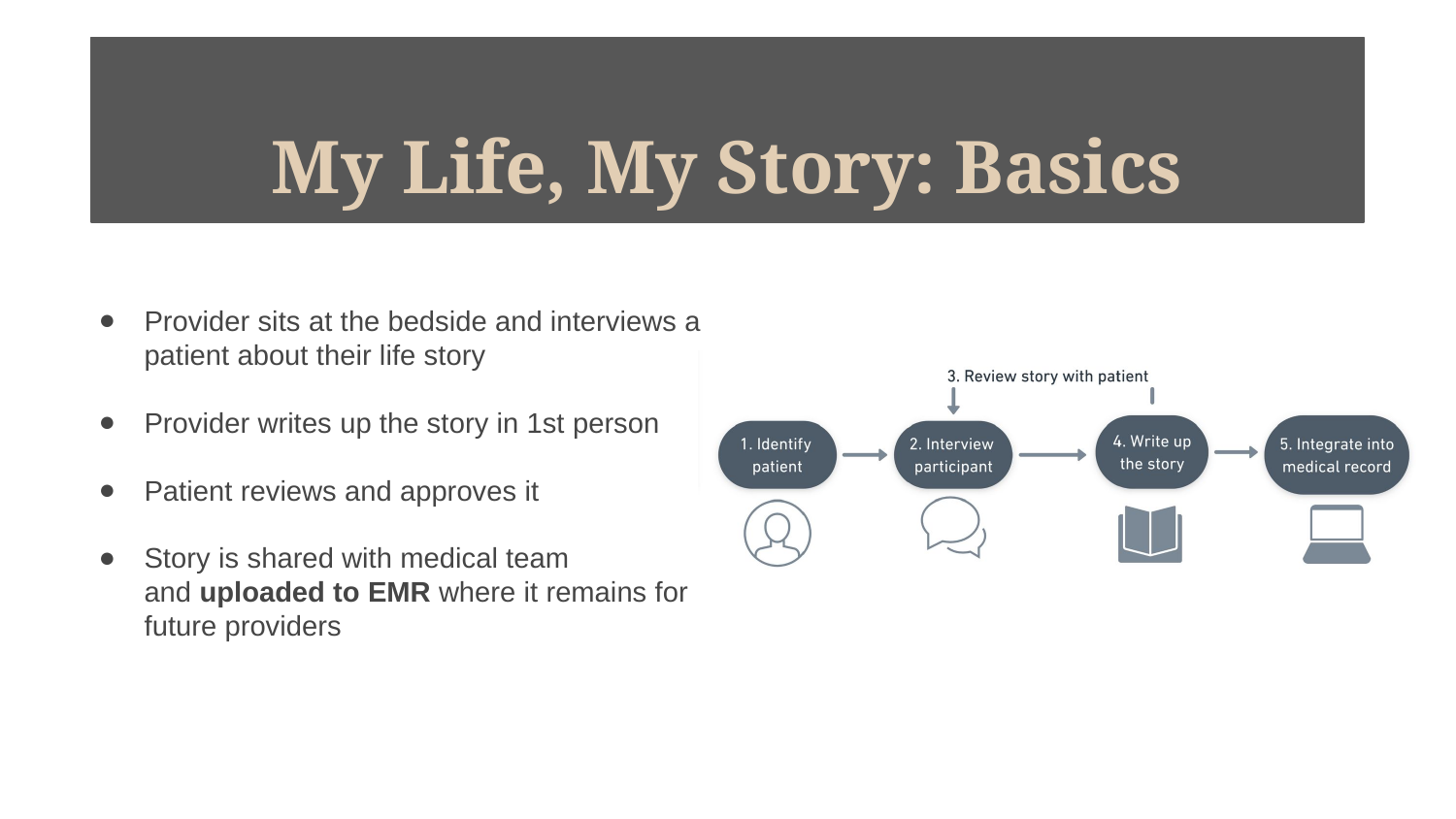

My Life, My Story: Basics
Provider sits at the bedside and interviews a patient about their life story
Provider writes up the story in 1st person
Patient reviews and approves it
Story is shared with medical team
and uploaded to EMR where it remains for future providers

## Slide 4
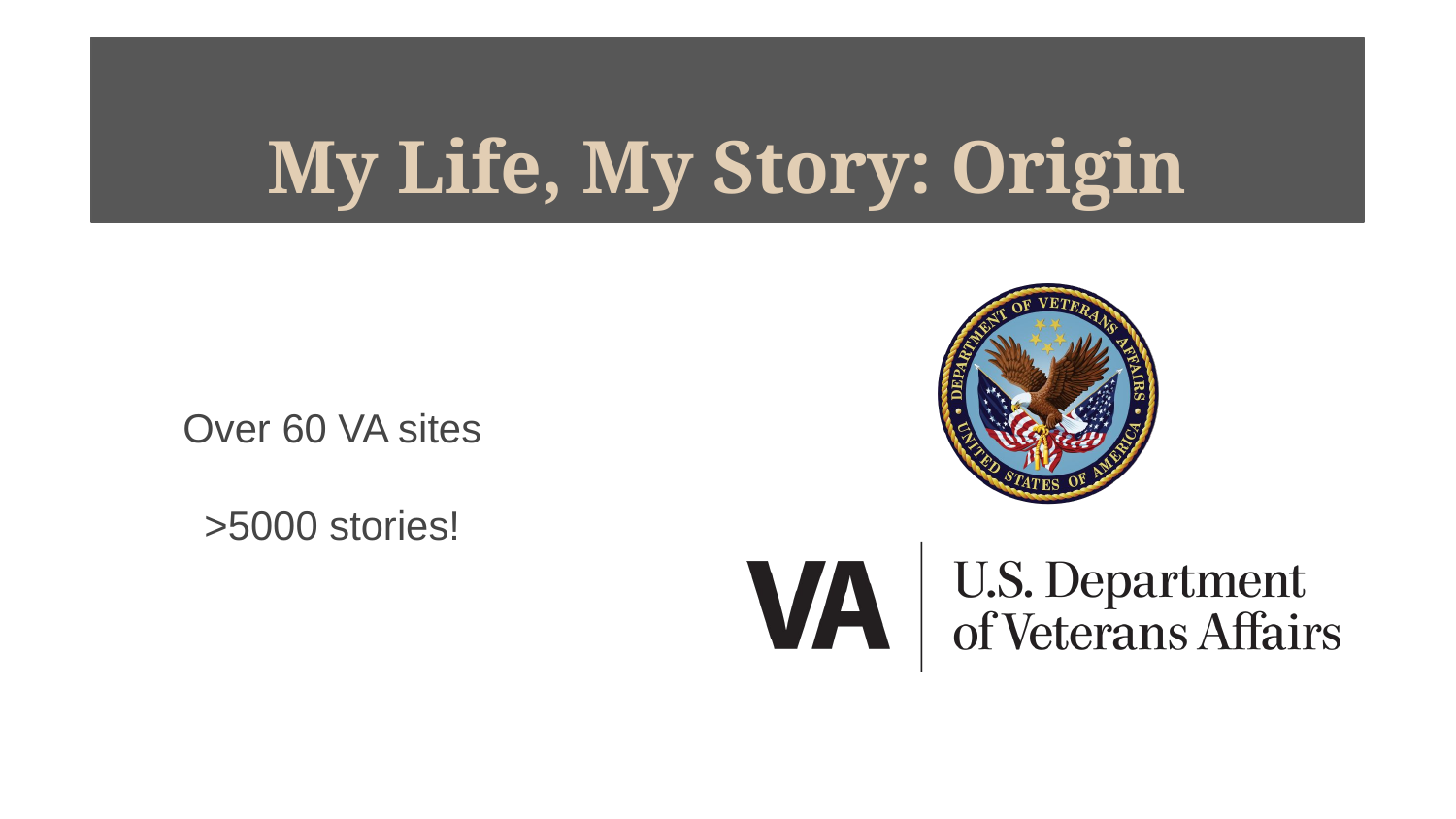

My Life, My Story: Origin
Over 60 VA sites
>5000 stories!

## Slide 5
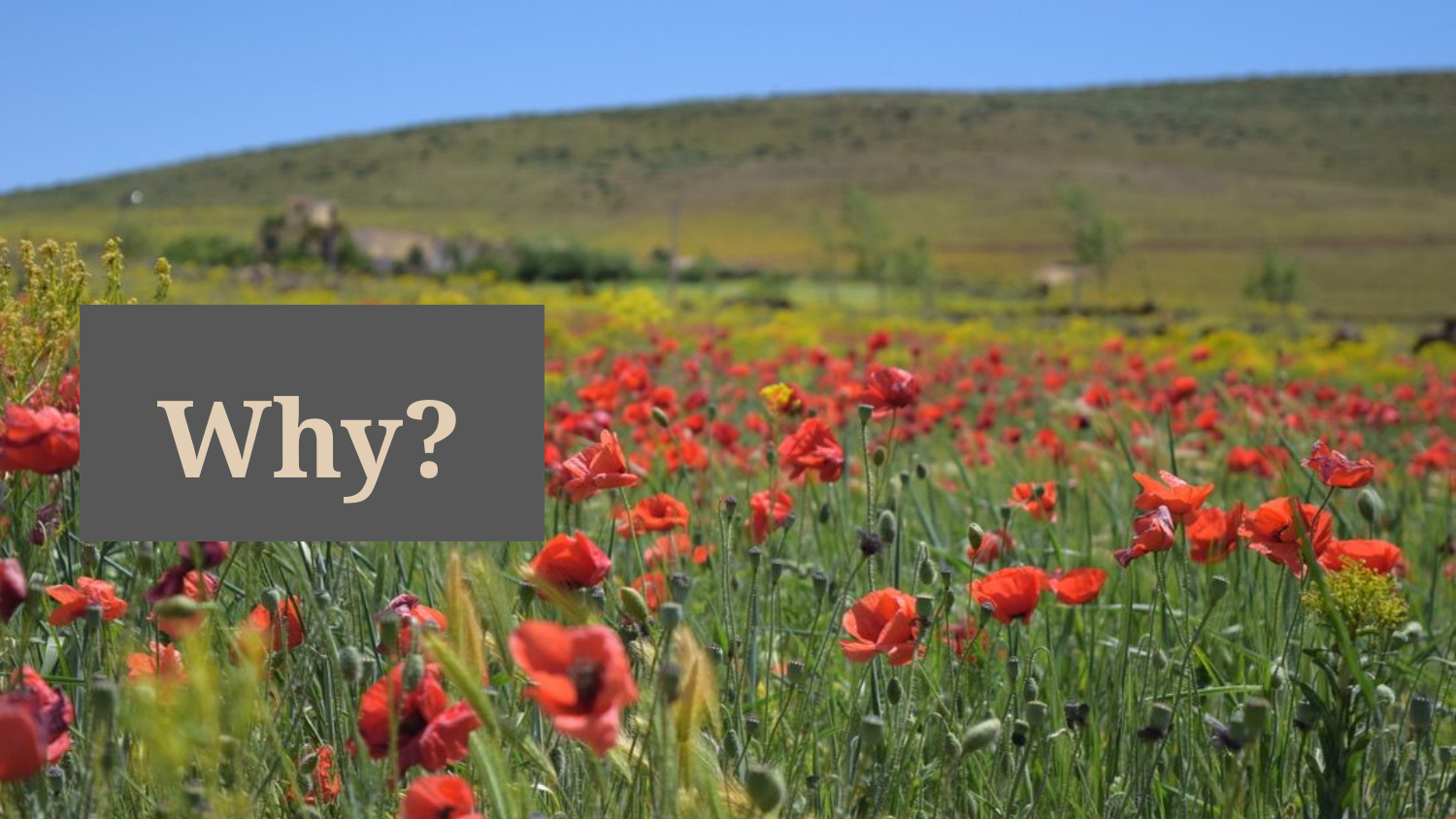

Why?

## Slide 6
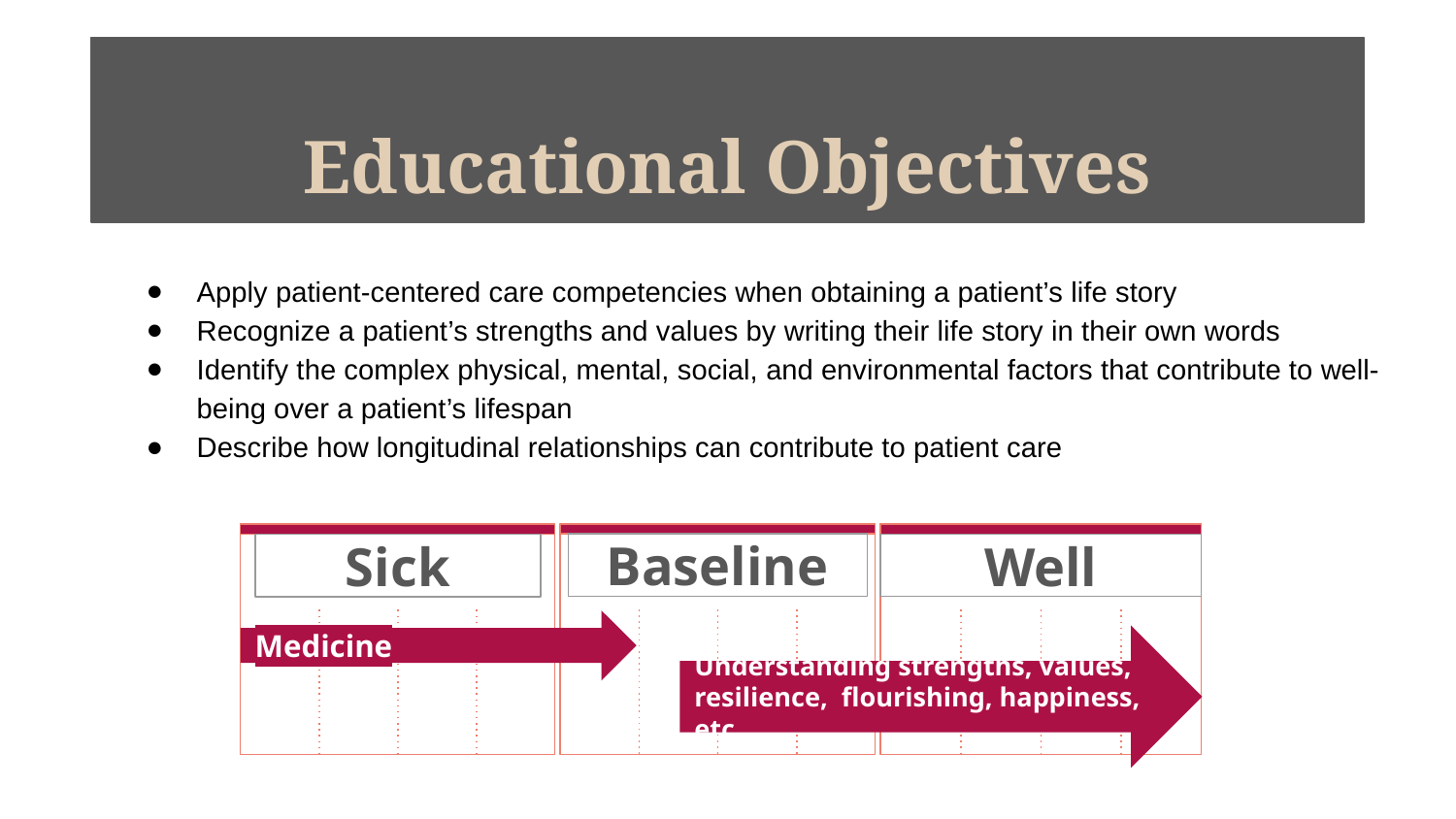

Educational Objectives
Apply patient-centered care competencies when obtaining a patient’s life story
Recognize a patient’s strengths and values by writing their life story in their own words
Identify the complex physical, mental, social, and environmental factors that contribute to well-being over a patient’s lifespan
Describe how longitudinal relationships can contribute to patient care
Baseline
Well
Sick
Medicine
Understanding strengths, values, resilience, flourishing, happiness, etc.

## Slide 7
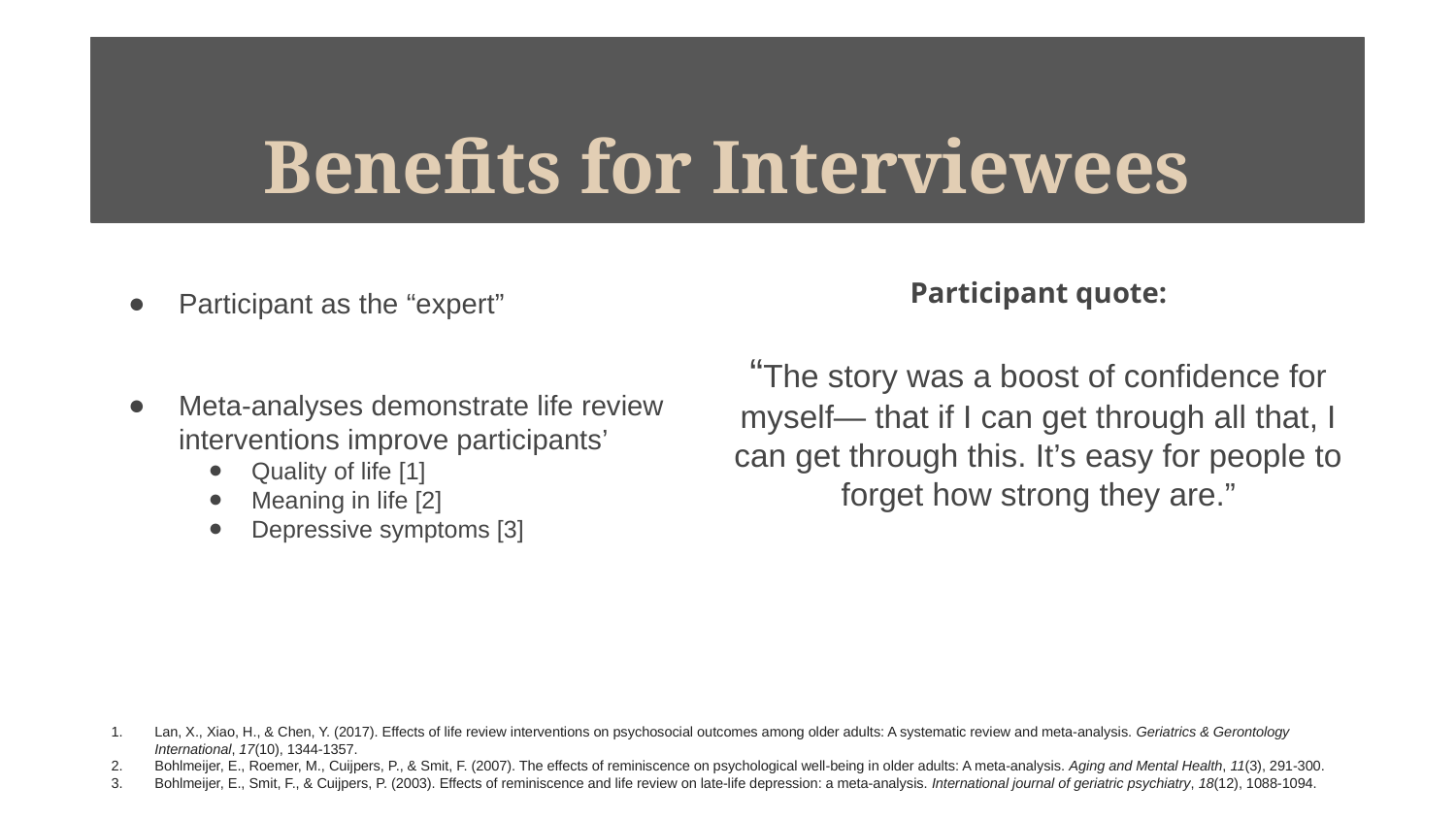

Benefits for Interviewees
Participant quote:
“The story was a boost of confidence for myself— that if I can get through all that, I can get through this. It’s easy for people to forget how strong they are.”
Participant as the “expert”
Meta-analyses demonstrate life review interventions improve participants’
Quality of life [1]
Meaning in life [2]
Depressive symptoms [3]
Lan, X., Xiao, H., & Chen, Y. (2017). Effects of life review interventions on psychosocial outcomes among older adults: A systematic review and meta‐analysis. Geriatrics & Gerontology International, 17(10), 1344-1357.
Bohlmeijer, E., Roemer, M., Cuijpers, P., & Smit, F. (2007). The effects of reminiscence on psychological well-being in older adults: A meta-analysis. Aging and Mental Health, 11(3), 291-300.
Bohlmeijer, E., Smit, F., & Cuijpers, P. (2003). Effects of reminiscence and life review on late‐life depression: a meta‐analysis. International journal of geriatric psychiatry, 18(12), 1088-1094.

## Slide 8
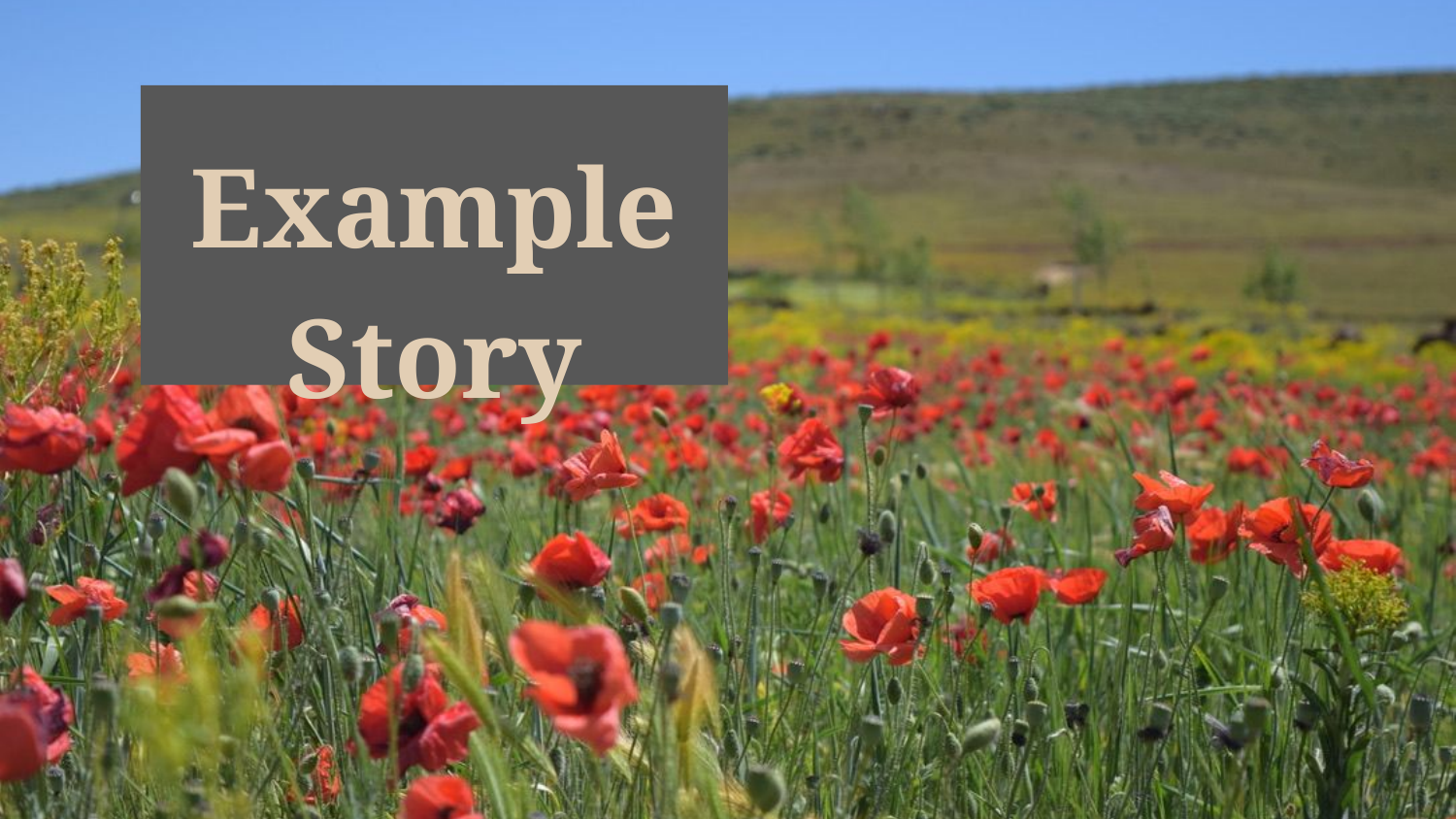

Example Story

## Slide 9
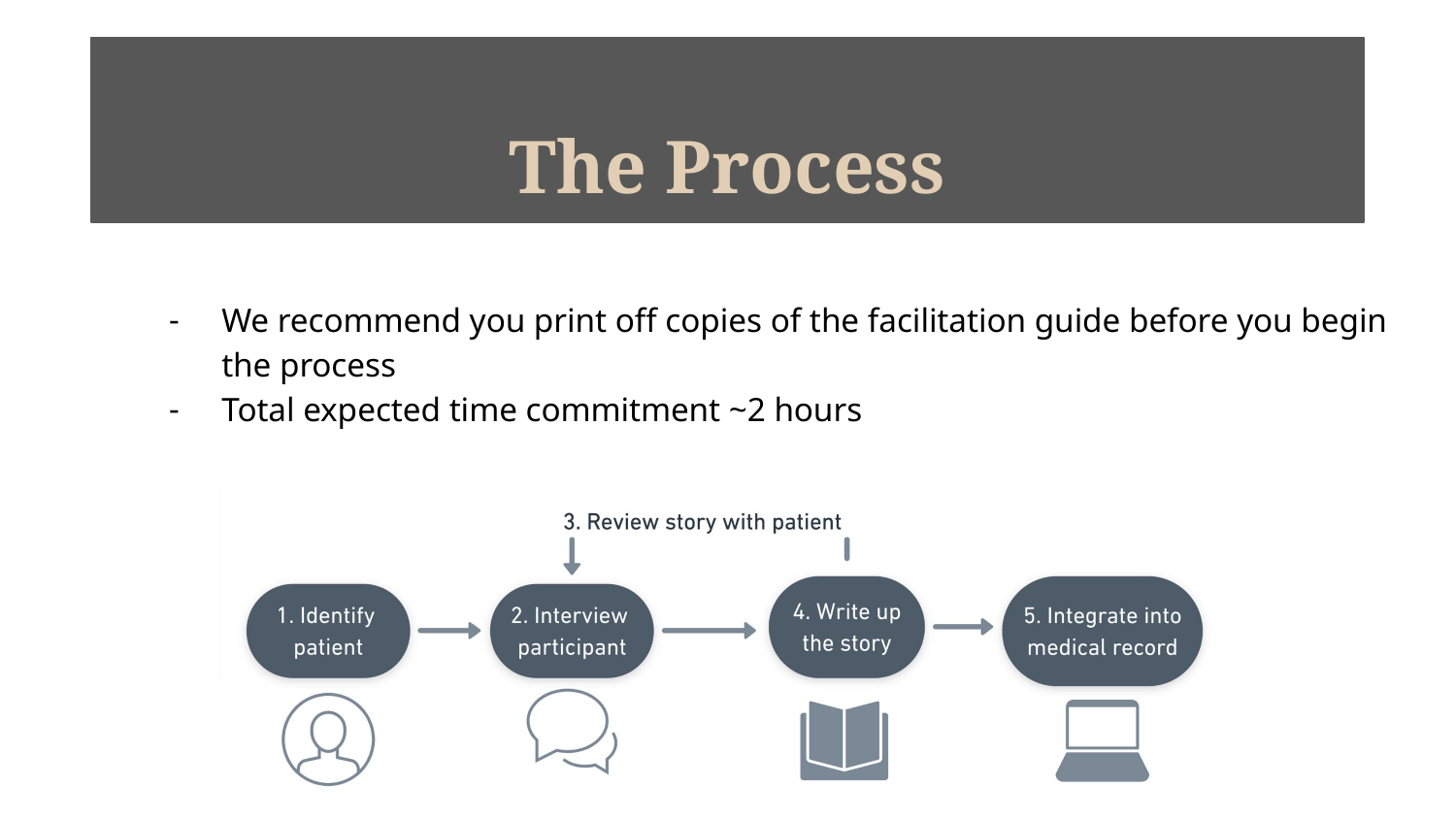

The Process
We recommend you print off copies of the facilitation guide before you begin the process
Total expected time commitment ~2 hours

## Slide 10
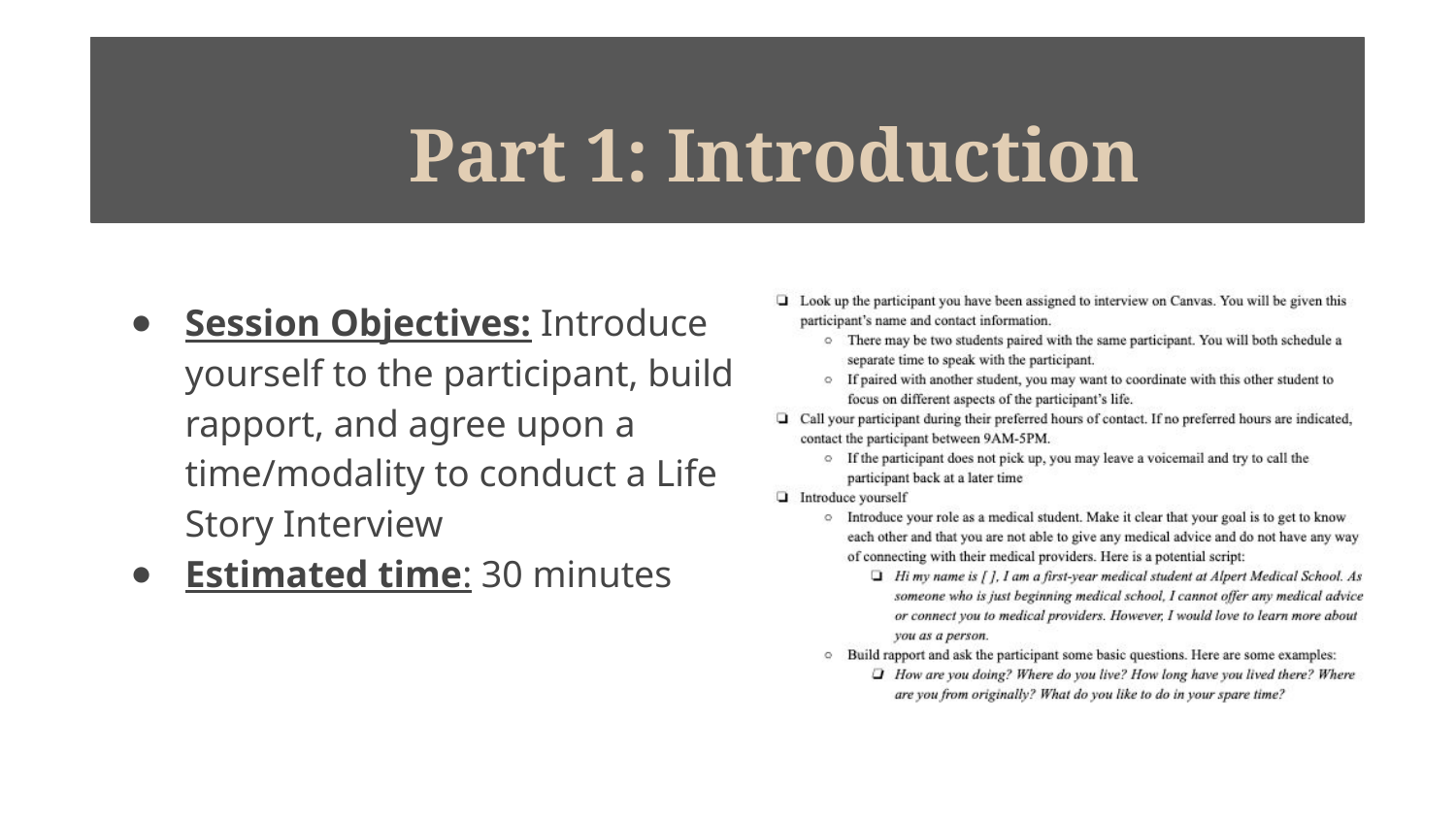

Part 1: Introduction
Session Objectives: Introduce yourself to the participant, build rapport, and agree upon a time/modality to conduct a Life Story Interview
Estimated time: 30 minutes

## Slide 11
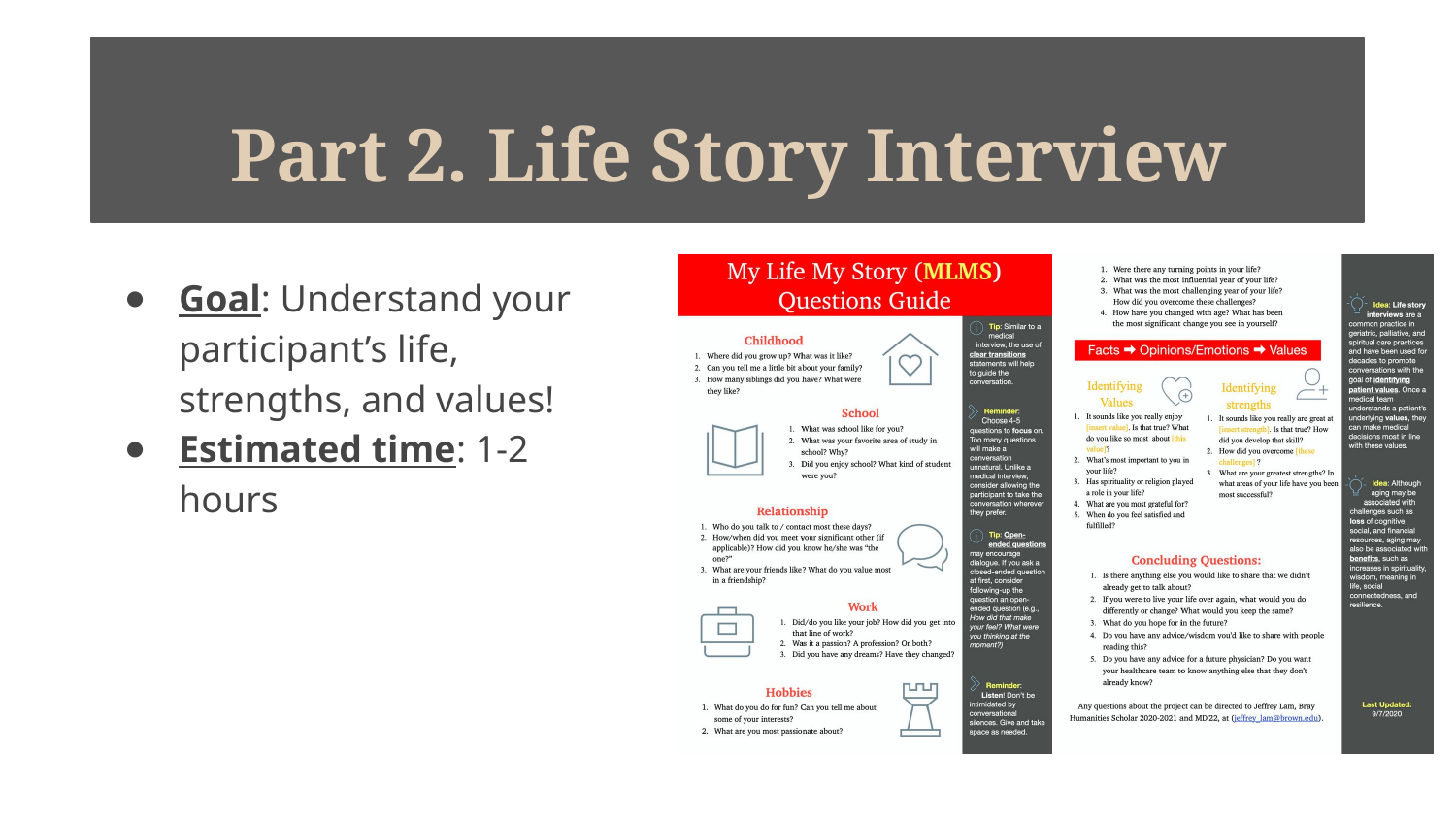

Part 2. Life Story Interview
Goal: Understand your participant’s life, strengths, and values!
Estimated time: 1-2 hours

## Slide 12
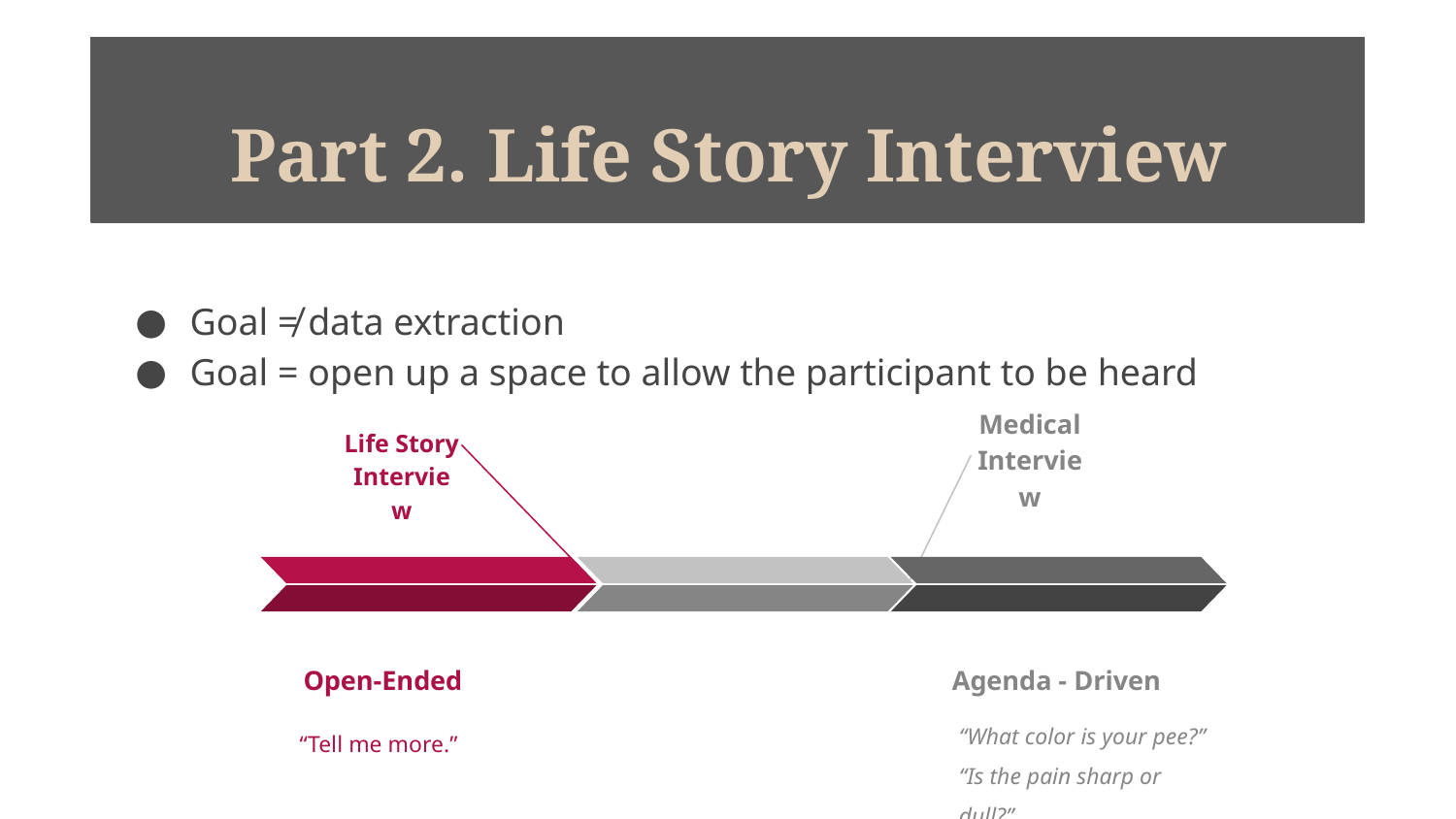

Part 2. Life Story Interview
Goal ≠ data extraction
Goal = open up a space to allow the participant to be heard
Medical Interview
Life Story Interview
Open-Ended
“Tell me more.”
Agenda - Driven
“What color is your pee?”
“Is the pain sharp or dull?”

## Slide 13
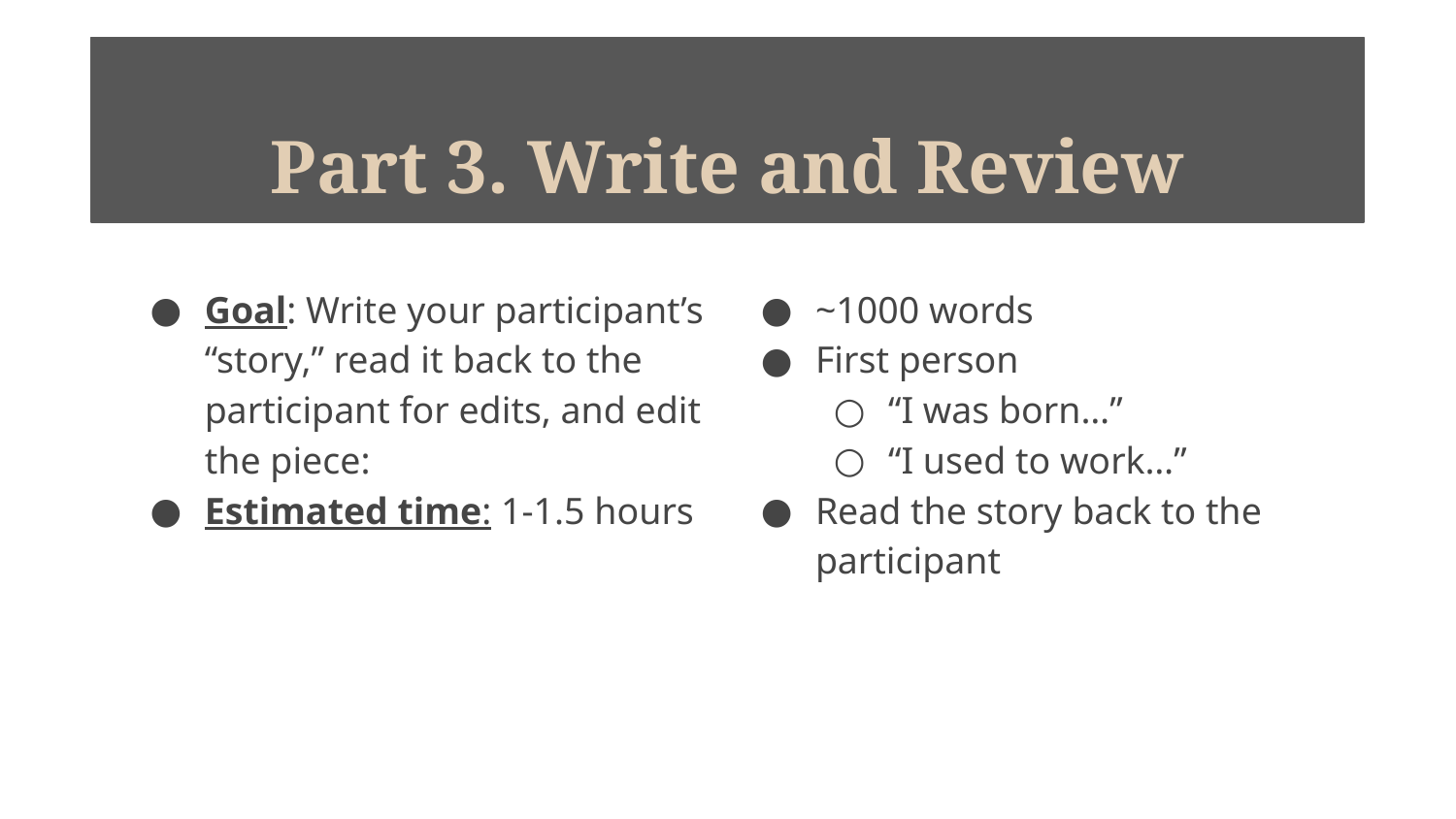

Part 3. Write and Review
Goal: Write your participant’s “story,” read it back to the participant for edits, and edit the piece:
Estimated time: 1-1.5 hours
~1000 words
First person
“I was born…”
“I used to work…”
Read the story back to the participant

## Slide 14
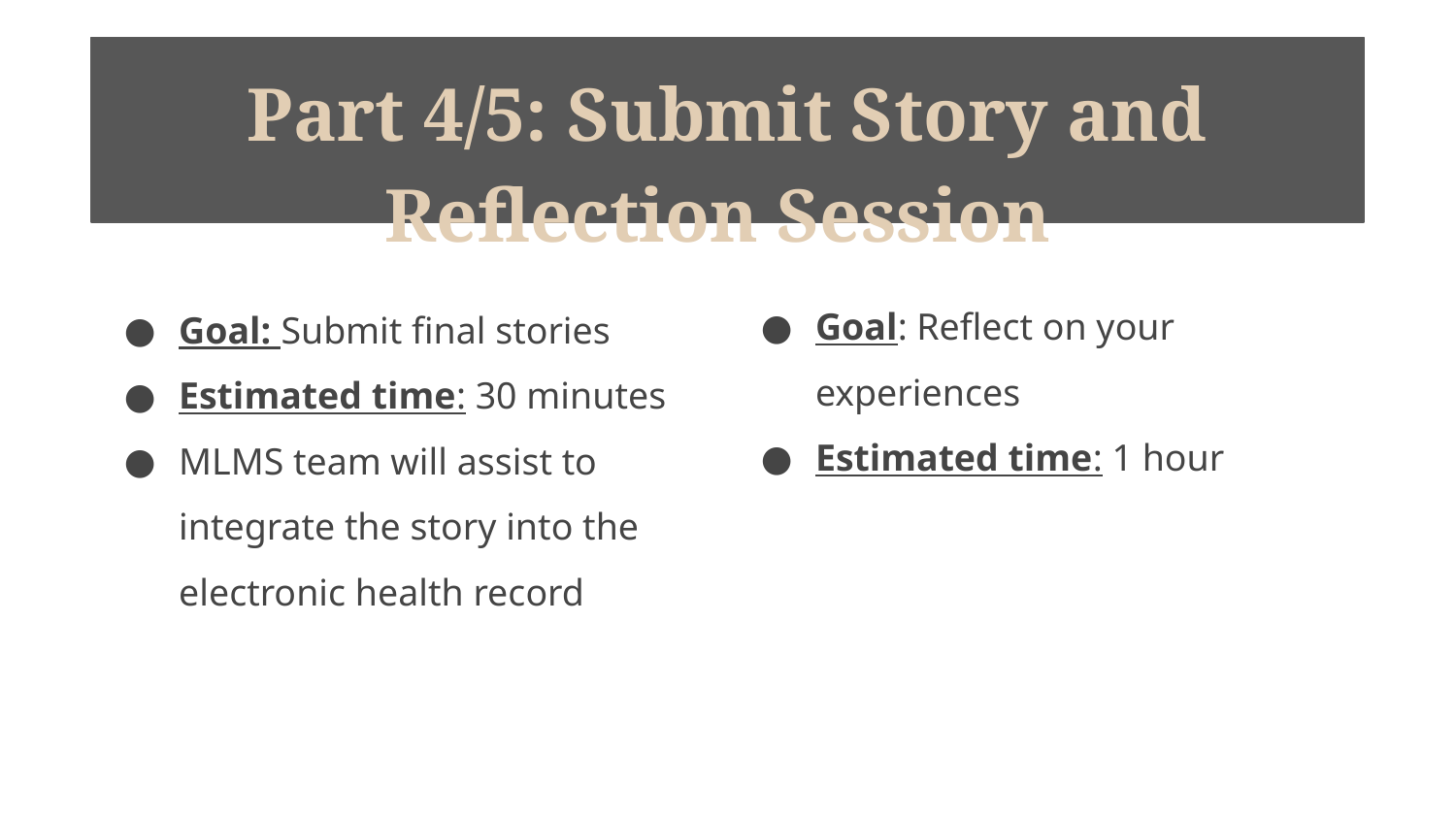

Part 4/5: Submit Story and Reflection Session
Goal: Reflect on your experiences
Estimated time: 1 hour
Goal: Submit final stories
Estimated time: 30 minutes
MLMS team will assist to integrate the story into the electronic health record

## Slide 15
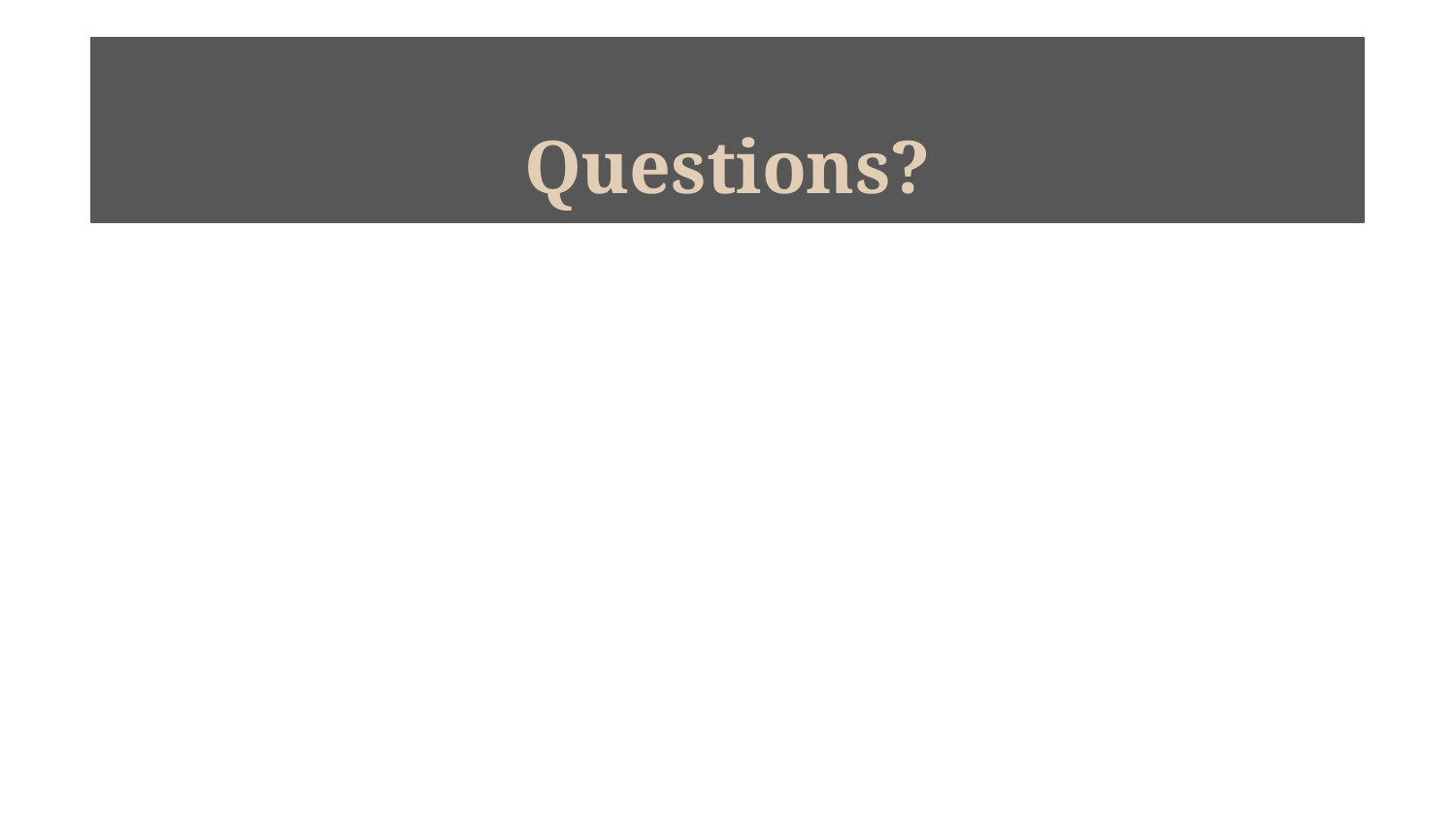

Questions?
