# Supplementary material for: My Life, My Story: Integrating a Life Story Narrative Component Into Medical Student Curricula
Source: MedEdPORTAL. 2022 Jan 26;18:11211. doi: 10.15766/mep_2374-8265.11211 (PMC8789965; doi:10.15766/mep_2374-8265.11211)
Supplement: Supplementary file 1 — PowerPoint Presentation.pptxPreclinical Facilitation Guide.docxClinical Facilitation Guide.docxSurvey Instruments.docx [file mep_2374-8265.11211-s001.zip › B. Preclinical Facilitation Guide.docx]

# My Life, My Story: Preclinical Guide

**What is My Life, My Story?**

My Life My Story (MLMS) is a project that was started by a psychiatry resident in a (Veteran’s affair) VA hospital in Madison, Wisconsin, and since, the project has spread across the country. The goal of the initiative is to get medical trainees back to the bedside and actively listen to our patients’ stories by allowing time for and creating a structure for medical trainees to better understand and empathize with patients as whole human-beings.


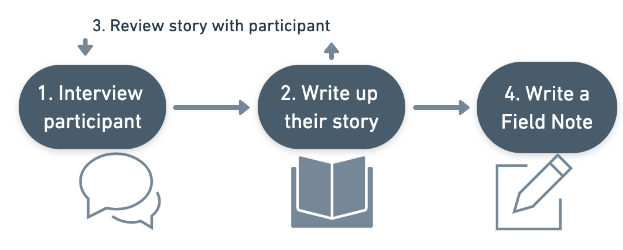


Image: Author Owned

Each first year medical student will complete a My Life, My Story interview and write-up with an volunteer living in the community as their service-learning activity this semester. These adults were recruited from various community organizations.

At the introductory training session, students will be given a facilitation guide, which contains a script for asking the volunteer’s permission, suggested interview questions, tips on active listening, and instructions for writing up the story. This session constitutes the required preparation for service-learning.

Students will subsequently be asked to complete a field note reflection on their experience. The field note prompt can be found in Part Four of this guide.

Students will also send their narratives to their Doctoring small group faculty. At the debrief session, students will be asked to reflect on their experiences with each other and share what they learned with their Doctoring small group.

**Goals of *My Life, My Story: Resiliency Conversations* Curriculum**

1. Apply patient-centered care competencies when obtaining a patient’s life story
2. List a patient’s strengths and values by writing their life story in their own words
3. Identify the complex physical, mental, social, and environmental factors that contribute to well-being over a patient’s lifespan
4. Describe how longitudinal relationships can contribute to patient care
5. Practice empathic, non-judgmental listening skills

| My Life, My Story Table of Contents |
| --- |
| 1. [Introduction to participant and scheduling an interview](#_l9ymign1d17s) |
| 1. [Conduct a virtual life story interview with participant](#_mnch34p5dwfo) |
| 1. [Write your participant’s story, read it back to the participant for edits](#_ldx8hwtd7wav) |
| 1. [Reflection and Field Note](#_xhsxvl2ygcih) |
| - [Logistical and Troubleshooting Tips](#_11njbowr7d60) - [Further information and opportunities](#_ddyqgfofzr7q) |

#

# Part 1: Introduction to Participant

**Session Objectives:** Introduce yourself to the participant, build rapport, and agree upon a time to conduct a Life Story History

**Estimated time**: 1 hour

- We recruited from a diverse group of community agencies.. All participants received an introductory letter explaining the program and gave verbal consent to participate.
- Look up the participant you have been assigned to interview. You will be given this participant’s name and contact information.
  - There may be two students paired with the same participant. You will both schedule a separate time to speak with the participant.
- Call your participant during their preferred hours of contact. If no preferred hours are indicated, contact the participant between 9AM-5PM.
  - If the participant does not pick up, you may leave a voicemail and try to call the participant back at a later time
- Introduce yourself
  - Introduce your role as a medical student. Make it clear that your goal is to get to know each other and that you are not able to give any medical advice and do not have any way of connecting with their medical providers. Here is a potential script:
    - *Hi my name is [ ], I am a first-year medical student calling because you as part of the My Life, My Story program that you had expressed interest in through [community agency] earlier. As someone who is just beginning medical school, I cannot offer any medical advice or connect you to medical providers. However, I would love to learn more about you as a person.*
    - **In your introductory phone call with your partnered volunteer, please reference the agency that referred them to participate in My Life, My Story so that they understand why you are calling.
    - ****Make sure that the volunteer knows whom they are speaking to and what the conversation is about. Speak clearly and be patient. You may have to repeat yourself several times during the conversation.
  - Build rapport and ask the participant some basic questions. Here are some examples:
    - *How are you doing? Where do you live? How long have you lived there? Where are you from originally? What do you like to do in your spare time?*
  - *Self-disclosure and boundary setting*
    - Throughout this introduction, you may consider sharing some information about yourself with your participant. Sharing some facts about yourself can help your interviewee trust you and feel connected.
- After you have built some rapport with the participant, ask the participant if they would like to participate in a life story interview next week. Here is a verbal consent script you may use:
  - *I am wondering if we could schedule a time next week to ask you more questions about your life. As a medical student, I think it is important that we get to know and understand the community members whom we take care of. This interview would be part of a larger project called My Life, My Story that is done at many hospitals across the country, and that we are starting. Your participation would involve us doing an interview with you to learn more about your life and your values. At any point during the process, if you decide you are no longer interested in participating, that is fine. My overall goal is to learn more about you as a person and the important stories of your life. Afterwards, I would like to write up your life story and we could review it together the following week. Would you be interested in taking part next week?”*
- **Schedule a time next week to conduct the life story interview**
  - Discuss the possibility of completing the interview on FaceTime or Zoom, if the participant is interested and comfortable with these forms of communication.
  - Some agencies requested that you inform an agency liaison of interview appointment times so they can remind the volunteers.

#

# Part 2: Conduct a Life Story Interview

**Goal**: Understand the events of your participant’s life, with the overall goal of identifying their current values and strengths

**Estimated time**: 1-2 hours

**Suggested Preparation:** Read this article on resilience and watch this Ted Talk on narrative humility

- Moran M. Resilience, No Depression Best Predict Successful Aging. Psychiatric News. https://psychnews.psychiatryonline.org/doi/full/10.1176/appi.pn.2013.1a13. Published January 4, 2013. Accessed May 14, 2021.
- TEDxTalks. Narrative Humility: Sayantani DasGupta at TEDxSLC. YouTube. https://www.youtube.com/watch?v=gZ3ucjmcZwY. Published July 10, 2013. Accessed May 14, 2021.
- Contact your resident/participant at the time you scheduled the previous week
  - This may be through a phone call, FaceTime, or Zoom
- Set the agenda for the day. You may use the following script:
  - *“As we discussed last week,* *I am wondering if you would still be willing to participate in an interview to learn more about your life and values. At any point during the process, if you decide you are no longer interested in participating, please let me know. My overall goal is to learn more about you as a person and the important stories of your life. Are you still interested in taking part?”*
- Start your interview. You may move chronologically through the participant’s life or skip around.
  - Below is a conversation guide with a list of suggested questions if you get stuck, or run out of questions to ask.
  - The volunteer population includes a diverse group of individuals. Their education level and experiences may vary widely. If the volunteer doesn't understand a question, you may need to phrase it another way.
- After you have asked your questions, be sure to ask the participant if there is anything else they would like to share.
- Schedule a follow-up time next week to review the story you’ve drafted.
- Thank them for sharing their story with you.
- If you have any clinical concerns about the participant or have a difficult time getting in touch with your assigned volunteer, please contact the MLMS team.

###

###
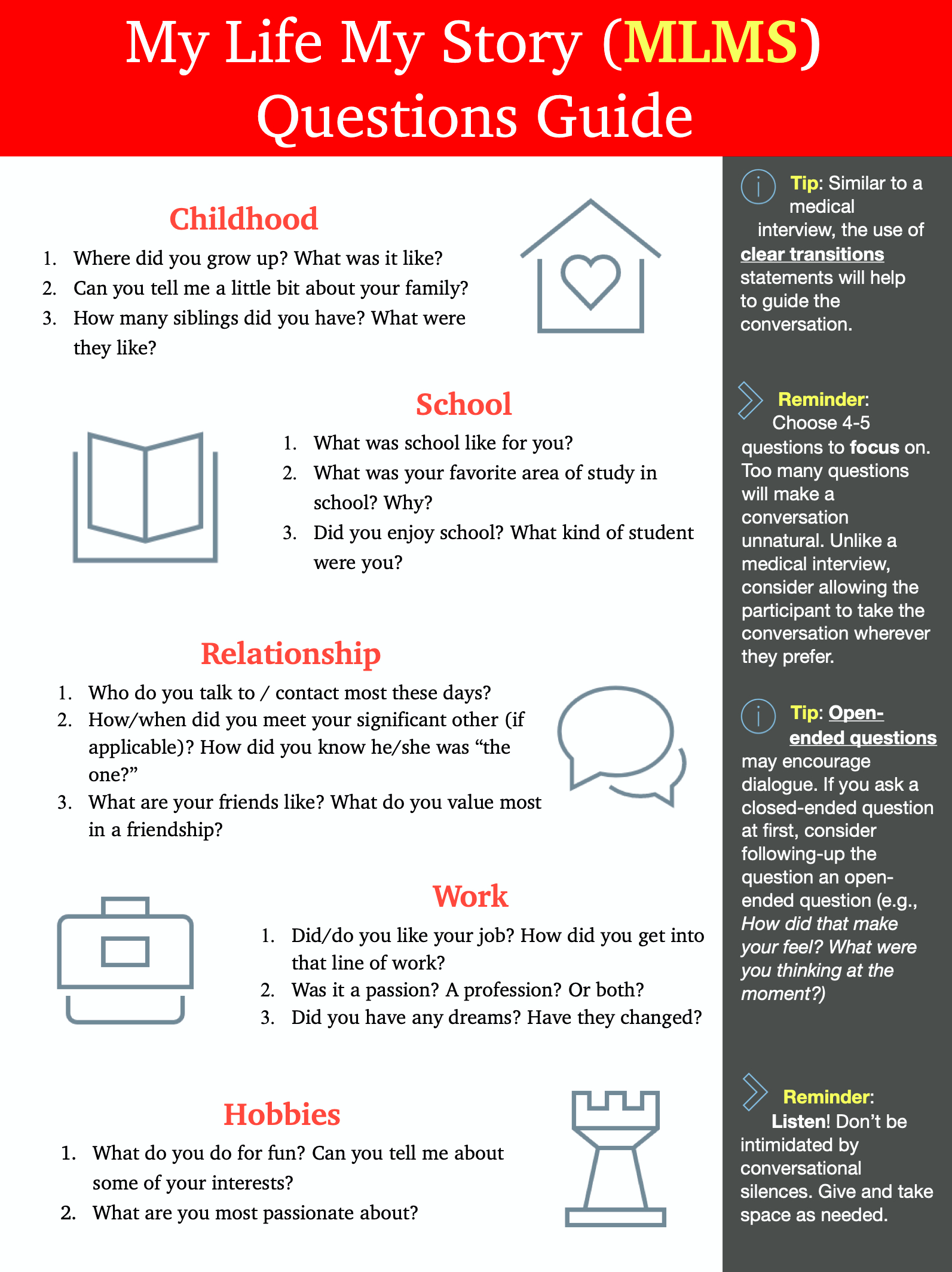


Image: Author Owned

###
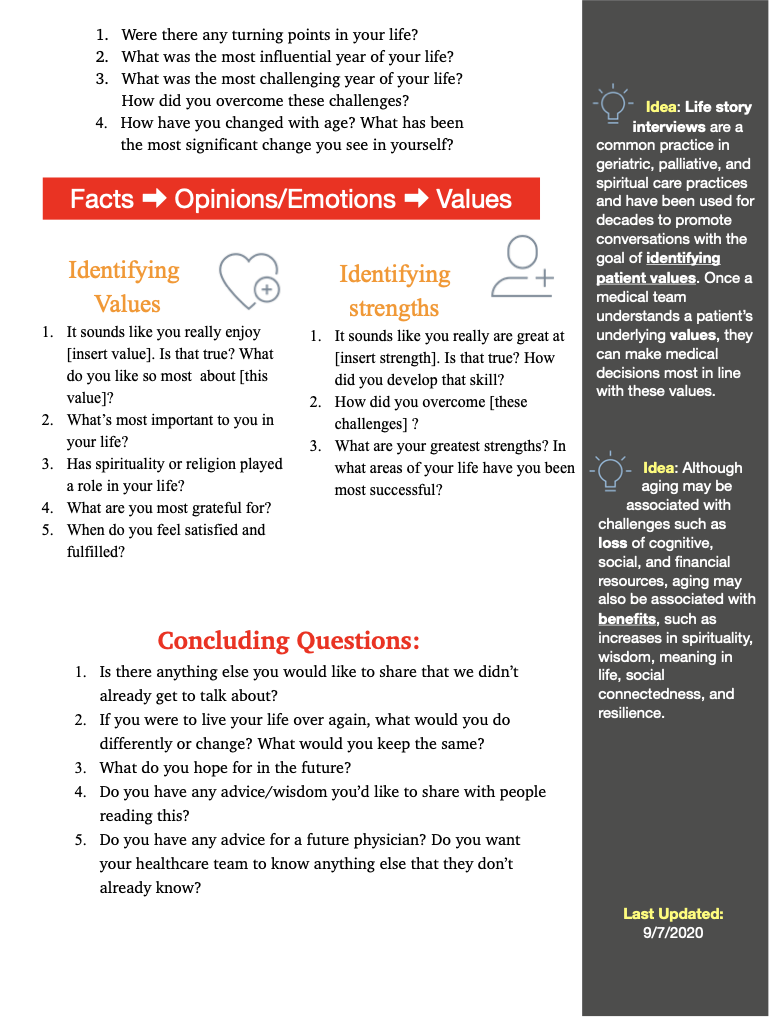


Image: Author Owned

# Part 3: Write and Review

**Goal**: Write your volunteer’s “story,” read it back to the participant for edits, and edit the piece:

**Estimated time**: 1-1.5 hours

**Suggested Preparation:** Read this article on resilience and watch this Ted Talk on narrative humility

- Moran M. Resilience, No Depression Best Predict Successful Aging. Psychiatric News. https://psychnews.psychiatryonline.org/doi/full/10.1176/appi.pn.2013.1a13. Published January 4, 2013. Accessed May 14, 2021.
- TEDxTalks. Narrative Humility: Sayantani DasGupta at TEDxSLC. YouTube. https://www.youtube.com/watch?v=gZ3ucjmcZwY. Published July 10, 2013. Accessed May 14, 2021.
- Write up the interview in first person (“I was born…” “I used to work…”) using the participant’s own words. Please try to limit the narrative to about 1000 words (1-2 pages).
  - **Note: While writing in first person may feel awkward at first, we ask you to write in first person because previous students have noted this allows them to better put themselves in the participant’s shoes and write in the participant’s own words. Furthermore, this is the one of the only times in medicine you will write in first person, rather than third person. This is also the only opportunity for a patient to have their own voice in the medical record.
- Contact the participant again at the time you scheduled last week
- Read the story back to the participant for any edits and final approval before finalizing the story.
  - *I drafted a story and I was wondering if I could read it aloud to you so that you can make any edits and add any details I might have missed?*
- Offer to print or email copies for the participant and/or their family.
- Obtain verbal consent to share this story with your Doctoring faculty and MLMS team
  - *“Would it be okay if I shared this with my professor and some of my classmates so we can learn from the stories of community members? I could not include your name or any identifying information, if that makes you more comfortable.*
- Ask the participant if there is anything else he or she would like to share.
- Offer to share a copy of the story with the participant. Discuss if the participant prefers the postal service or email. If mailing, the student can use the school address for a return address
- Thank the participant for sharing their story with you and their time.

##

# Part 4: Reflection and Field Note

**Goal**: Reflect on your experience

**Estimated time**: 1 hour

**Please write a 1-page written reflection using the following prompt:**

- Complete reflective “Field Note” using the following prompt: Resilient people tend to use their personal strengths to combat adversity and/or seek support from others. These strengths or forms of support are different for different people. Identify the forms of strength or resiliency the participant demonstrated during their life. What values and themes did you identify? How did this interview experience impact your perspective on patient resiliency? What did you learn from your interview experience that can inform your future practice as a physician in terms of delivering patient centered, trauma informed, inclusive care?
- Send the final story to your faculty.

**Debrief Questions for Faculty:**

1. Would anyone like to share a quote from their story? Or a salient moment you had while interviewing the participant?
2. What values and themes did you identify in your participant’s story? How did this interview experience impact your perspective on the patient?
3. What surprised you about this experience?
4. What parts of it were most enjoyable? Were any parts awkward or uncomfortable?
5. How was this different from other patient interactions?
6. What are your takeaways from this experience? Did you learn anything about yourself? About the way you want to practice medicine?

#

##

##

## Logistical and Troubleshooting Tips

**Things to know**

- We recruited from a diverse group of community agencies.
- In your introductory phone call with your partnered volunteer, please reference the agency that referred them to participate in My Life, My Story so that they understand why you are calling.
- Make sure that the volunteer knows whom they are speaking to and what the conversation is about. Speak clearly and be patient. You may have to repeat yourself several times during the conversation.
- The volunteer population includes a diverse group of individuals. Their education level and experiences may vary widely. If the volunteer doesn't understand a question, you may need to phrase it another way.
- If you have a difficult time getting in touch with your assigned volunteer, please contact the MLMS team

**Things to consider**

- If the volunteer expresses a concern you think should be followed up on, for e.g., they express a medical or safety concern, please contact our team
- Offer to share a copy of the story with the participant. Discuss if the participant prefers the postal service or email.

**Troubleshooting tips**

If you have a hard time understanding the volunteer or if they have a hard time understanding you:

- Is it a volume issue?
- Are you or the volunteer speaking directly into your phone or device microphone?
- Limit background noise and call from a quiet location.
- If the background noise is in the volunteers environment, ask if they have a TV or radio on, and ask if they can turn it off or go to a quieter location.

If the volunteer reports a difficult time understanding you:

- Ask them if they have any hearing concerns?
- If yes, ask if they wear hearing aids and are they currently using them?
- See this article on tips for talking to someone with hearing loss:
  - Tips To Talking On The Phone When Someone Has Hearing Loss. https://bayaudiology.co.nz/blog/tips-to-talking-on-the-phone-when-someone-has-hearing-loss. Accessed May 14, 2021.

## Further Information and Opportunities

1. More information on My Life, My Story
   1. Sable-Smith B. Storytelling Helps Hospital Staff Discover The Person Within The Patient. NPR. https://www.npr.org/sections/health-shots/2019/06/08/729351842/storytelling-helps-hospital-staff-discover-the-person-within-the-patient. Published June 8, 2019. Accessed May 13, 2021.
   2. An Introduction to My Life My Story. YouTube. https://www.youtube.com/watch?v=Fe6WAm2Xbuk. Published June 28, 2018. Accessed May 13, 2021.
   3. Feingold-Link M. A Bear in the Woods. Journal of Medical Education and Curricular Development. 2020;7:238212051989939. doi:10.1177/2382120519899391
   4. Nathan S, Fiore LL, Saunders S, et al. My life, my story: Teaching patient centered care competencies for older adults through life story work. Gerontology & Geriatrics Education. 2019:1-14. doi:10.1080/02701960.2019.1665038
2. More information on understanding patient strengths and values in medicine
   1. Moran M. Resilience, No Depression Best Predict Successful Aging. Psychiatric News. https://psychnews.psychiatryonline.org/doi/full/10.1176/appi.pn.2013.1a13. Published January 4, 2013. Accessed May 14, 2021.
   2. VanderWeele TJ. On the promotion of human flourishing. Proceedings of the National Academy of Sciences. 2017;114(31):8148-8156. doi:10.1073/pnas.1702996114
   3. Jeste DV, Palmer BW, Rettew DC, Boardman S. Positive Psychiatry. The Journal of Clinical Psychiatry. 2015;76(06):675-683. doi:10.4088/jcp.14nr09599
3. Narrative medicine resources and selected publications
   1. TEDxTalks. Narrative Humility: Sayantani DasGupta at TEDxSLC. YouTube. https://www.youtube.com/watch?v=gZ3ucjmcZwY. Published July 10, 2013. Accessed May 14, 2021.
   2. Krisberg K. Narrative Medicine: Every Patient Has a Story. AAMC. https://www.aamc.org/news-insights/narrative-medicine-every-patient-has-story. Published March 28, 2017. Accessed May 13, 2021.
   3. Charon R. Narrative Medicine. JAMA. 2001;286(15):1897. doi:10.1001/jama.286.15.1897
