# Supplementary material for: My Life, My Story: Integrating a Life Story Narrative Component Into Medical Student Curricula
Source: MedEdPORTAL. 2022 Jan 26;18:11211. doi: 10.15766/mep_2374-8265.11211 (PMC8789965; doi:10.15766/mep_2374-8265.11211)
Supplement: Supplementary file 1 — PowerPoint Presentation.pptxPreclinical Facilitation Guide.docxClinical Facilitation Guide.docxSurvey Instruments.docx [file mep_2374-8265.11211-s001.zip › C. Clinical Facilitation Guide.docx]

My Life, My Story: Clinical Guide

Are you curious about what your patient was like when they were younger? Have you ever wished you had more time to learn about a patient's story beyond their medical one-liner? Do you want to *really* have a chance to get to know your patients?

**
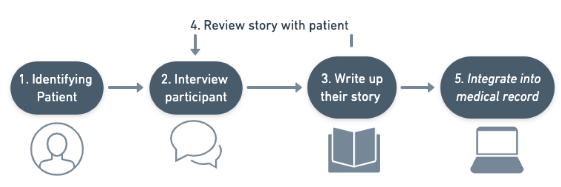
**

Image: Author Owned

| My Life, My Story Table of Contents |
| --- |
| 1. [Before the interview: Identifying patient and asking for consent](#_59ha8a8mho0i) 2. [Interviewing the participant](#_jb7lur800oqh) 3. [After the interview: Writing and Reviewing](#_ttyxx4grwnt) 4. [Submit final story and integrate into EHR](#_x3nlsq3k5q8s) |
| - [Further information and opportunities](#_m7ckpcb57ap2) |

### Goals of *My Life, My Story*

1. Apply patient-centered care competencies when obtaining a patient’s life story
2. List a patient’s strengths and values by writing their life story in their own words
3. Identify the complex physical, mental, social, and environmental factors that contribute to well-being over a patient’s lifespan
4. Describe how longitudinal relationships can contribute to patient care
5. Practice empathic, non-judgmental listening skills

## Instructions:

This process involves a bedside or phone interview with a patient (approximately 30-45 minutes), typing up the narrative, reading the story back to the patient for edits, editing the piece, printing copies for the patient, asking the patient some demographic questions, sending the story to the My Life, My Story team, and completing a quick feedback form. The total time commitment is expected to be ~2 hours.

### Before the Interview

1. **Select a patient**
   1. Identify a patient to participate; anyone who says, “Yes” is perfect. About 50% of people who are offered will decline. It is impossible to predict without offering.
      1. Tips for identifying a patient:
         1. You can offer these interviews to any patient, including patients you are not carrying or patients on other care teams.
         2. Ask your intern/resident. They will likely be familiar with MLMS interviews and might have potential patients in mind.
         3. If you are still having trouble identifying a patient, please contact the MLMS team to assist.
   2. Note: if you are a native speaker of a language other than English, we highly encourage you to interview a patient in that language. If English is your only language, we encourage you to use the hospital interpreter service to interview patients who have limited English proficiency.
2. **Ask the patient if they would like to participate.** Here is one potential script:
   1. *I was wondering if you’d like to participate in a program aiming to allow medical professionals to get to know their patients better?* [If you have met the patient before, you could state a reason why you would like to learn about this person more.] *This is a project that is done at many hospitals across the country, and that we are starting here. Your participation would involve us doing an interview with you to learn more about you outside of just the medical things. The goal is for us to know more about your life and who you are as a person. At any point during the process, if you decide you are no longer interested in participating, that is fine too. Your story and health information will remain confidential and stored securely within our health system and shared only with your medical providers and the My Life, My Story staff. Our goal is to eventually upload this story into your medical record for future providers to read! Again, our goal is to learn more about you as a person and the important stories of your life. Would you be interested in taking part?”*

### **Interview the patient!**

- 1. Feel free to use the Life Story Interview Guide to help guide your interview if you would like suggestions for questions, get stuck, or run out of questions to ask.

### After the Interview

1. **Ask the patient if there is anything else** they would like to share. Thank them for sharing their story with you.
2. **Write up the interview in first person** (“I was born…” “I used to work…”) using the patient’s own words in a note draft in your electronic medical record or Microsoft Word on a secure computer (a hospital computer or your laptop if it has been secured by IT to handle patient data). Please try to limit the narrative to about 1000 words (1-2 pages).
3. **Read the story back to the patient for any edits and final approval** before finalizing the story. Some patients don’t want to hear it back, but you should always offer. Here is one potential script:
   1. *I drafted a story and I was wondering if I could* [read it aloud to you/ask you to read the story] *so that you can make any edits and add any details I might have missed?*
4. **Offer to print copies for the patients and/or their family.** They can have as many copies as they would like.
5. **Obtain verbal consent to share this story** with their medical providers, the MLMS staff, and to put it in their medical record. Here is one potential script:
   1. *Would you be comfortable if I were to share this with the MLMS staff and put this story into your medical record for your future healthcare providers to see?*

### **Submit final story (and MLMS team will integrate into EHR)**

- 1. **Note: After you submit the stories to the MLMS team, we will review the story and integrate it into the medical record.

1. If you would like to debrief the interview for any reason, please reach out to the MLMS team. If you have any clinical concerns about the patient, please address the primary clinical team.

###
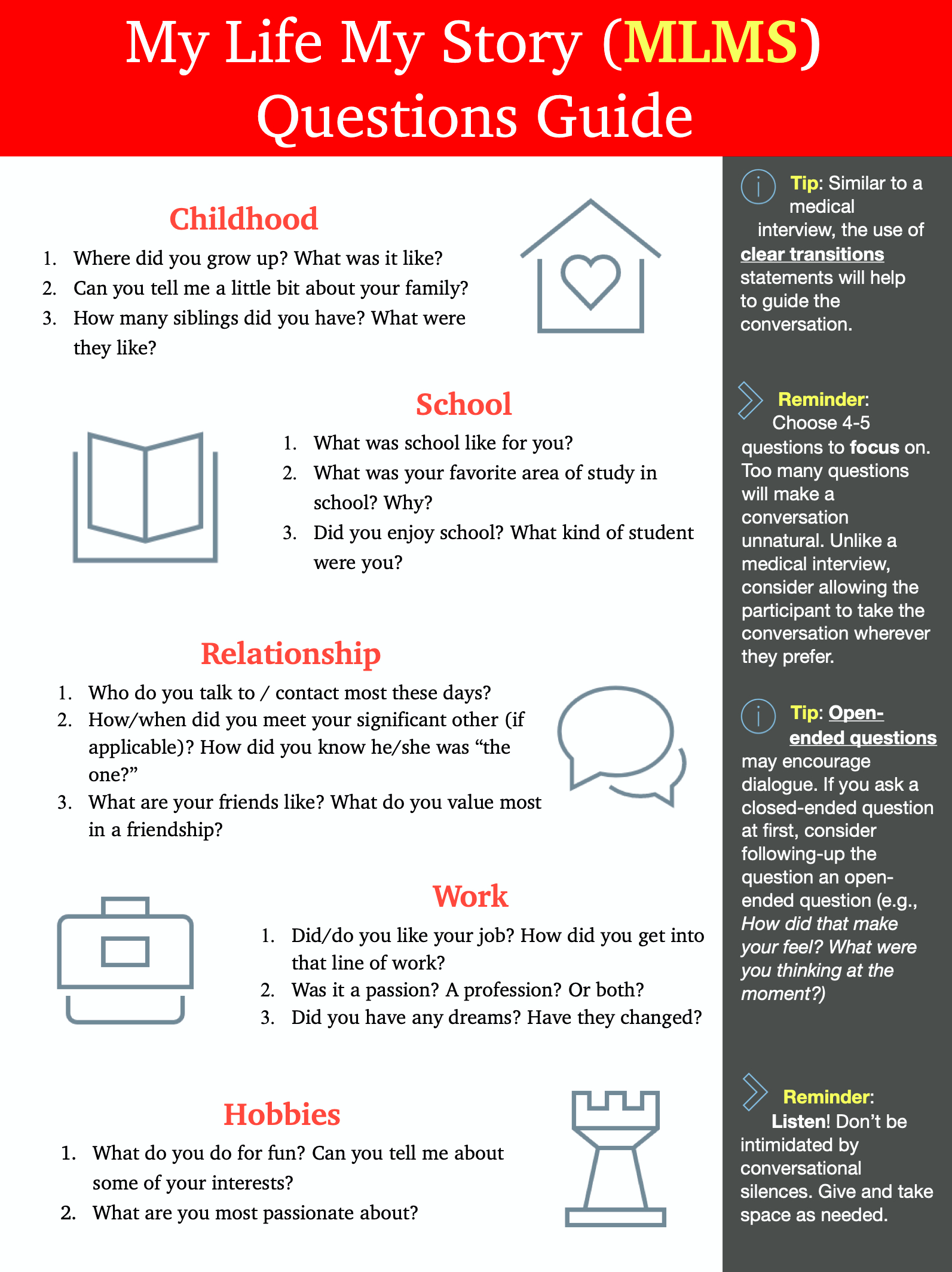


Image: Author Owned

###
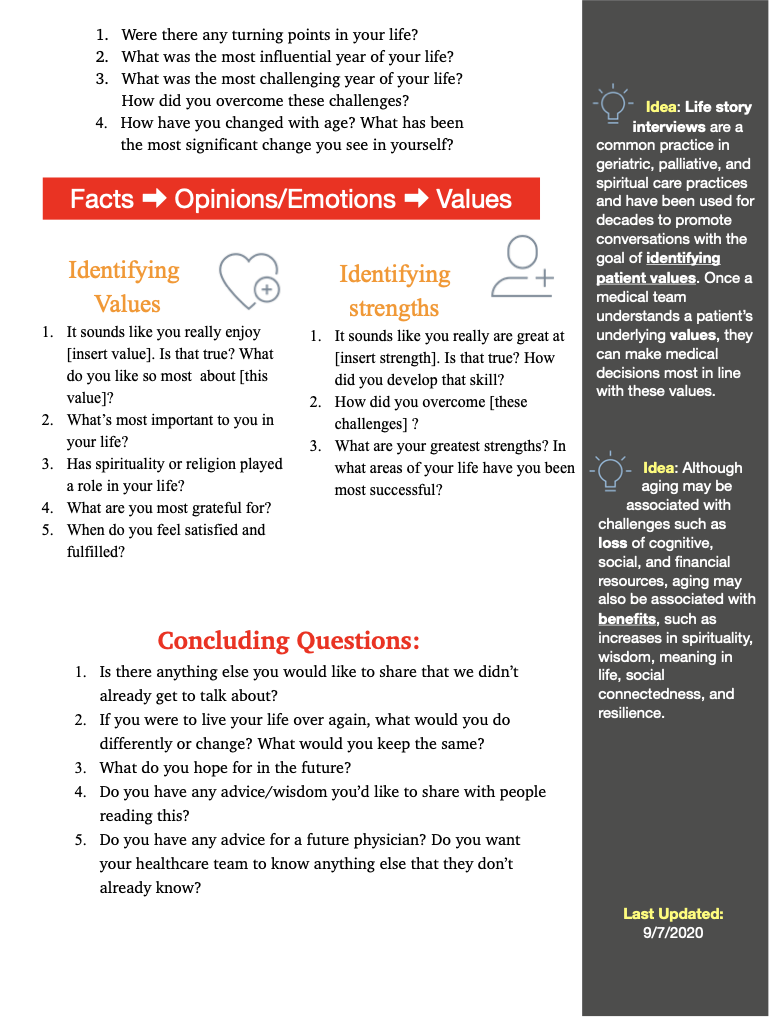


Image: Author Owned

## Debrief Questions for Reflection Session

1. Would anyone like to share a quote from their story? Or a salient moment you had while interviewing the participant?
2. What values and themes did you identify in your participant’s story? How did this interview experience impact your perspective on the patient?
3. What surprised you about this experience?
4. What parts of it were most enjoyable? Were any parts awkward or uncomfortable?
5. How was this different from other patient interactions?
6. What are your takeaways from this experience? Did you learn anything about yourself? About the way you want to practice medicine?

## Further Information and Opportunities

1. More information on My Life, My Story
   1. Sable-Smith B. Storytelling Helps Hospital Staff Discover The Person Within The Patient. NPR. https://www.npr.org/sections/health-shots/2019/06/08/729351842/storytelling-helps-hospital-staff-discover-the-person-within-the-patient. Published June 8, 2019. Accessed May 13, 2021.
   2. An Introduction to My Life My Story. YouTube. https://www.youtube.com/watch?v=Fe6WAm2Xbuk. Published June 28, 2018. Accessed May 13, 2021.
   3. Feingold-Link M. A Bear in the Woods. Journal of Medical Education and Curricular Development. 2020;7:238212051989939. doi:10.1177/2382120519899391
   4. Nathan S, Fiore LL, Saunders S, et al. My life, my story: Teaching patient centered care competencies for older adults through life story work. Gerontology & Geriatrics Education. 2019:1-14. doi:10.1080/02701960.2019.1665038
2. More information on understanding patient strengths and values in medicine
   1. Moran M. Resilience, No Depression Best Predict Successful Aging. Psychiatric News. https://psychnews.psychiatryonline.org/doi/full/10.1176/appi.pn.2013.1a13. Published January 4, 2013. Accessed May 14, 2021.
   2. VanderWeele TJ. On the promotion of human flourishing. Proceedings of the National Academy of Sciences. 2017;114(31):8148-8156. doi:10.1073/pnas.1702996114
   3. Jeste DV, Palmer BW, Rettew DC, Boardman S. Positive Psychiatry. The Journal of Clinical Psychiatry. 2015;76(06):675-683. doi:10.4088/jcp.14nr09599
3. Narrative medicine resources and selected publications
   1. TEDxTalks. Narrative Humility: Sayantani DasGupta at TEDxSLC. YouTube. https://www.youtube.com/watch?v=gZ3ucjmcZwY. Published July 10, 2013. Accessed May 14, 2021.
   2. Krisberg K. Narrative Medicine: Every Patient Has a Story. AAMC. https://www.aamc.org/news-insights/narrative-medicine-every-patient-has-story. Published March 28, 2017. Accessed May 13, 2021.
   3. Charon R. Narrative Medicine. JAMA. 2001;286(15):1897. doi:10.1001/jama.286.15.1897
