# Supplementary material for: My Life, My Story: Integrating a Life Story Narrative Component Into Medical Student Curricula
Source: MedEdPORTAL. 2022 Jan 26;18:11211. doi: 10.15766/mep_2374-8265.11211 (PMC8789965; doi:10.15766/mep_2374-8265.11211)
Supplement: Supplementary file 1 — PowerPoint Presentation.pptxPreclinical Facilitation Guide.docxClinical Facilitation Guide.docxSurvey Instruments.docx [file mep_2374-8265.11211-s001.zip › D. Survey Instruments.docx]

**My Life, My Story Baseline Assessment**

Q1 What is your email address?

________________________________________________________________

Q2 Affiliation

- MS1
- MS2
- MS3
- MS4
- Other

Q3 What specialty are you interested in?

________________________________________________________________

Q4 What is your age?

________________________________________________________________

Q5 What is your gender identity?

- Male
- Female
- Other

Q6 How would you currently self-rate these following skills:

|  | Poor | Fair | Good | Very Good | Excellent |
| --- | --- | --- | --- | --- | --- |
| Recognize the patient's thoughts and feelings |  |  |  |  |  |
| Be attentive and responsive |  |  |  |  |  |
| Treat the patient in a caring manner |  |  |  |  |  |
| Make the patient experience me as empathetic |  |  |  |  |  |
| Show a genuine interest in the patient and his/her situation |  |  |  |  |  |

**My Life, My Story Post-Intervention Assessment**

You can print a copy of this form.

Q1 What is your email address?

________________________________________________________________

Q2 Affiliation

- MS1
- MS2
- MS3
- MS4
- Other

Q3 What is your age?

________________________________________________________________

Q4 What is your gender identity?

- Male
- Female
- Other

Q5 Did you take part in a training session?

- Yes, I took part in an Life Story Review training session
- No, I received information about the project over email
- Other ________________________________________________

Q6 How many Life Story Interviews have you completed in total (including your most recent interview)?

- 0
- 1
- 2
- ≥3

Q7 Approximately how many minutes did you spend interviewing the patient for the most recent interview?

________________________________________________________________

Q8 Did you use one of the facilitation guides during your conversation?

- No, I had an unguided conversation/ I let the patient direct the conversation
- Yes, I used the Life Story Interview guide
- Other

Q9 How would you currently self-rate these following skills:

|  | Poor | Fair | Good | Very Good | Excellent |
| --- | --- | --- | --- | --- | --- |
| Recognize the patient's thoughts and feelings |  |  |  |  |  |
| Be attentive and responsive |  |  |  |  |  |
| Treat the patient in a caring manner |  |  |  |  |  |
| Make the patient experience me as empathetic |  |  |  |  |  |
| Show a genuine interest in the patient and his/her situation |  |  |  |  |  |

Q10 Please rate how much doing this project has changed your ability to...

|  | Not effective at all | Slightly effective | Moderately effective | Very effective | Extremely effective |
| --- | --- | --- | --- | --- | --- |
| Recognize the patient's thoughts and feelings |  |  |  |  |  |
| Be attentive and responsive |  |  |  |  |  |
| Treat the patient in a caring manner |  |  |  |  |  |
| Make the patient experience me as empathetic |  |  |  |  |  |
| Show a genuine interest in the patient and his/her situation |  |  |  |  |  |

Q11 Please rate your agreement with the following statements

|  | Strongly disagree | Somewhat disagree | Neither agree nor disagree | Somewhat agree | Strongly agree |
| --- | --- | --- | --- | --- | --- |
| This project was a good use of my time |  |  |  |  |  |
| I found this experience valuable |  |  |  |  |  |
| This experience fostered more connection with patients |  |  |  |  |  |
| Having "whole patient-centered" conversations may improve patient care |  |  |  |  |  |
| This project offered an experience not provided elsewhere in my medical education |  |  |  |  |  |

Q12 How was your experience?

________________________________________________________________

Q13 What, if any, is the value of this project?

________________________________________________________________

Q14 Do you have any suggestions for how we could improve this project in the future?

________________________________________________________________
